# Technology-Supported Integrated Care Innovations to Support Diabetes and Mental Health Care: Scoping Review

**DOI:** 10.2196/44652

**Published:** 2023-05-09

**Authors:** Megan Racey, Carly Whitmore, Paige Alliston, Joseph A Cafazzo, Allison Crawford, David Castle, Rosa Dragonetti, Donna Fitzpatrick-Lewis, Milos Jovkovic, Osnat C Melamed, Farooq Naeem, Peter Senior, Gillian Strudwick, Seeta Ramdass, Victor Vien, Peter Selby, Diana Sherifali

**Affiliations:** 1 McMaster Evidence Review and Synthesis Team McMaster University Hamilton, ON Canada; 2 School of Nursing McMaster University Hamilton, ON Canada; 3 Centre for Addiction and Mental Health Toronto, ON Canada; 4 Healthcare Human Factors University Health Network Toronto, ON Canada; 5 eHealth Innovation University Health Network Toronto, ON Canada; 6 University of Toronto Toronto, ON Canada; 7 Department of Family and Community Medicine University of Toronto Toronto, ON Canada; 8 Clinical Islet Transplant Program University of Alberta Edmonton, AB Canada; 9 Department of Medicine Division of Endocrinology University of Alberta Edmonton, AB Canada; 10 Diabetes Action Canada Toronto, ON Canada; 11 McGill University Montreal, QC Canada

**Keywords:** technology, mental health, type 2 diabetes, type 1 diabetes, virtual care, integrated care, scoping review, health information technology, digital health, support, psychosocial, education, application, distress, clinical integration, intervention

## Abstract

**Background:**

For individuals living with diabetes and its psychosocial comorbidities (eg, depression, anxiety, and distress), there remains limited access to interprofessional, integrated care that includes mental health support, education, and follow-up. Health technology, broadly defined as the application of organized knowledge or skill as software, devices, and systems to solve health problems and improve quality of life, is emerging as a means of addressing these gaps. There is thus a need to understand how such technologies are being used to support, educate, and help individuals living with co-occurring diabetes and mental health distress or disorder.

**Objective:**

The purpose of this scoping review was to (1) describe the literature on technology-enabled integrated interventions for diabetes and mental health; (2) apply frameworks from the Mental Health Commission of Canada and World Health Organization to elucidate the components, type, processes, and users of technology-enabled integrated interventions for diabetes and mental health; and (3) map the level of integration of interventions for diabetes and mental health.

**Methods:**

We searched 6 databases from inception to February 2022 for English-language, peer-reviewed studies of any design or type that used technology to actively support both diabetes and any mental health distress or disorder in succession or concurrently among people with diabetes (type 1 diabetes, type 2 diabetes, and gestational diabetes). Reviewers screened citations and extracted data including study characteristics and details about the technology and integration used.

**Results:**

We included 24 studies described in 38 publications. These studies were conducted in a range of settings and sites of care including both web-based and in-person settings. Studies were mostly website-based (n=13) and used technology for wellness and prevention (n=16) and intervention and treatment (n=15). The primary users of these technologies were clients and health care providers. All the included intervention studies (n=20) used technology for clinical integration, but only 7 studies also used the technology for professional integration.

**Conclusions:**

The findings of this scoping review suggest that there is a growing body of literature on integrated care for diabetes and mental health enabled by technology. However, gaps still exist with how to best equip health care professionals with the knowledge and skills to offer integrated care. Future research is needed to continue to explore the purpose, level, and breadth of technology-enabled integration to facilitate an approach to overcome or address care fragmentation for diabetes and mental health and to understand how health technology can further drive the scale-up of innovative integrated interventions.

## Introduction

### Background

Every 1 in 10 globally and over 5 million Canadians are living with diagnosed diabetes today, including type 1 diabetes (T1D), type 2 diabetes (T2D), and gestational diabetes (GD); this number is steadily increasing worldwide [1]. Living with diabetes carries a heavy psychological burden [2] due to the constant need to adhere to regimented medication, diet, and exercise routines coupled with fear of diabetes complications, so much so that it has been said that diabetes is “one of the most psychologically demanding” chronic medical diseases [3]. Among individuals living with diabetes, the risk for developing mental illnesses such as depression and anxiety is greater than the general public [4]. Further, diabetes distress, defined as the negative emotions, despondency, and strain related to the burden of self-management, is increasingly prevalent [4-6]. The complexity of co-occurring mental and physical health challenges has contributed to increased calls for the integration of psychological care, such as cognitive behavioral therapy, into the care and management of diabetes [7-9]. However, despite this need, there remains limited access to interprofessional, integrated care that includes psychological support, education, and follow-up for individuals living with diabetes and associated comorbidities (eg, depression, anxiety, and distress).

Integrated care represents a solution to the fragmentation of care [10], such as the historically siloed physical and mental health care systems. Integrated care can take many forms, including the type of integration (eg, professional, clinical, or organizational), the level of integration (eg, macro-, meso-, or micro-), the breadth of integration (eg, a specific disease or a broader population group), and the intensity of integration (eg, informal linkages to aid in navigation or fully integrated whole systems) [10,11]. Although the literature examining integrated care as a concept continues to be developed and refined, there is a need to extend the use of the term to ultimately promote health and well-being and to support the development of enhanced skill and collaboration among health care providers. This includes understanding the ways that integrated care may address the adverse impacts of clinical complexity on individuals, their care experiences, and care outcomes, such as those found among individuals living with diabetes and mental health distress or disorder.

Emerging as a potential solution to this gap in clinical practice is the use of health technology as a means of offering person-centered, integrated diabetes and mental health care. Health technology is broadly defined as software, devices, and systems intended for the prevention, promotion, or rehabilitation of health, including those that assist, cure, or support [12]. These technologies are broad and include those categorized as eHealth or mobile health, wearable devices, and digital health. Although there is great variability in these technologies and how they are described, it is understood that these technologies should enable the processing and exchange of health information between end users such as patients and their care team using the internet [12]. Aligned with a rapid shift to remote care delivery in recent years, there is a need to understand how health technologies are being used to support, educate, and help manage individuals living with co-occurring diabetes and mental health distress or disorder.

### Study Objectives

The purpose of this scoping review was to (1) describe the literature on technology-enabled integrated interventions for diabetes and mental health; (2) apply frameworks from the Mental Health Commission of Canada and World Health Organization to elucidate the components, types, processes, and users of technology-enabled integrated interventions for diabetes and mental health; and (3) map the level of integration of these interventions for diabetes and mental health.

## Methods

### Study Design

This scoping review was guided by the methodologies of Arksey and O'Malley [13], Munn et al [14], and Pollock et al [15] and adheres to the PRISMA (Preferred Reporting Items for Systematic Reviews and Meta-Analyses) extension for scoping reviews [16].

### Search Strategy

The search terms, databases, and strategy were developed in consultation with a research librarian at McMaster University (Multimedia Appendix 1). We searched MEDLINE, Embase, Emcare, PsycINFO, Cochrane Database of Systematic Reviews, and Cochrane Central Register of Controlled Trials from inception to February 16, 2022. We manually searched reference lists of relevant reviews and included studies for citations that were not captured in our search. Duplicates were removed and citations were uploaded to a secure web-based platform for screening (DistillerSR; Evidence Partners Inc).

### Inclusion and Exclusion Criteria

Eligible studies of any design or type had to be published in English in a peer-reviewed journal and meet the following criteria: (1) report data on anyone with diabetes including T1D, T2D, and GD and (2) use technology (broadly defined, including the following: remote care, telephone, apps, SMS text messages, computer-based, or other e-technologies) to actively support both diabetes and any mental health distress or disorder in succession or concurrently. There were no criteria for the diagnosis of diabetes; however, studies with general adult populations or mixed populations, which provided subgroup analyses for participants with diabetes, were also considered for inclusion. We predefined that studies without subgroup analyses, but which had a mixed population, must have at least 80% of participants with our targeted condition to be included in our review. Participants did not need to have a mental health disorder at baseline for the study to be included provided the study aimed to address mental health concerns beyond merely as an outcome measurement. Outcomes were not used for inclusion or exclusion of studies. Studies were excluded if they (1) focused on either mental health or diabetes alone; (2) did not have explicit aims or components of the study that addressed both mental health and diabetes; (3) reported on data on those at-risk for, but not yet diagnosed with, diabetes; (4) were of diabetes-specific technology such as continuous blood glucose monitoring; and (5) were letters, commentaries, editorials, conference abstracts, or doctoral theses.

### Data Extraction and Charting

A team of researchers conducted the screening and data extraction (MR, MJ, PA, DS, DF-L, CW). A minimum of 2 reviewers were required to screen titles and abstracts of all potentially eligible studies independently and in duplicate. Articles marked for inclusion by either team member went on to full-text screening, which was completed independently and in duplicate by 2 team members and required consensus for inclusion or exclusion. After confirmation of the included studies, we looked for related publications that met our search dates and inclusion criteria and grouped publications that were based on the same study and intervention. We developed forms that were housed in a web-based systematic review software program. All authors provided feedback and approved the components of these forms. For each primary study, 1 team member extracted study characteristics (including the aim of the study, sample size, methods, population demographics, delivery person, study outcome types and time points, study length, location, and setting) and details about the technology following categories suggested by the Mental Health Commission of Canada: “Mental Health, Technology, and You” [17]; the Valentijn 2015 framework [18] in combination with its adapted version by Kaehne and Nies [19]; and the World Health Organization’s “Classification of Digital Health Interventions v1.0” [20]. Related or secondary publications were used to add details to the extracted data. Two team members independently verified all extracted data, and disagreements were resolved through discussion or third-party consultation. Conflicts were resolved by the lead researcher of this review (MR).

### Data Extraction Frameworks

Three frameworks were applied to organize and structure review findings specific to the purpose of technology in the intervention, the targeted primary users of the technology, and the level of integration that the technology advanced.

#### The Mental Health Commission of Canada’s “Mental Health, Technology, and You”

For each study, the purpose of using technologies was mapped against the definitions provided by the Mental Health Commission of Canada “Mental Health, Technology, and You” document [17]. This document contains 8 possible purposes of technology (wellness and prevention, coaching, peer-led support, intervention and treatment, web-based self-help, monitoring, crisis support, and recovery); we added a further two on the basis of the project team feedback (improve access to health care and digital literacy).

#### The World Health Organization’s “Classification of Digital Health Interventions v1.0”

Digital health intervention classifications were gathered using the World Health Organization’s “Classification of Digital Health Interventions v1.0” [20]. It provides groupings based on the targeted primary user. These groupings are as follows: (1) interventions for clients, (2) interventions for health care providers, (3) interventions for health system or resource managers, and (4) interventions for data services. Each grouping has subcomponents.

#### Valentijn 2015 Rainbow Model of Integrated Care

To describe the level of integration, we followed the Valentijn 2015 framework [18] in combination with its adapted version by Kaehne and Nies [19]. This framework introduces a taxonomy of integrated care, including 6 levels: clinical integration, professional integration, organizational integration, system integration, functional integration, and normative integration. For the purposes of this review, only clinical (ie, coordinating person-centered care across time, place, and discipline), professional (ie, interprofessional partnerships to deliver a continuum of care), organizational (ie, interorganizational relationships to deliver comprehensive services), and system integration (ie, coherent sets of rules and policies to facilitate both horizontal and vertical system integration) were explored.

### Ethical Considerations

As this study was solely literature based and did not involve any research participants or subjects, no formal ethics approval from the McMaster Research Ethics Board was required.

## Results

### Study Selection

**Figure 1:**
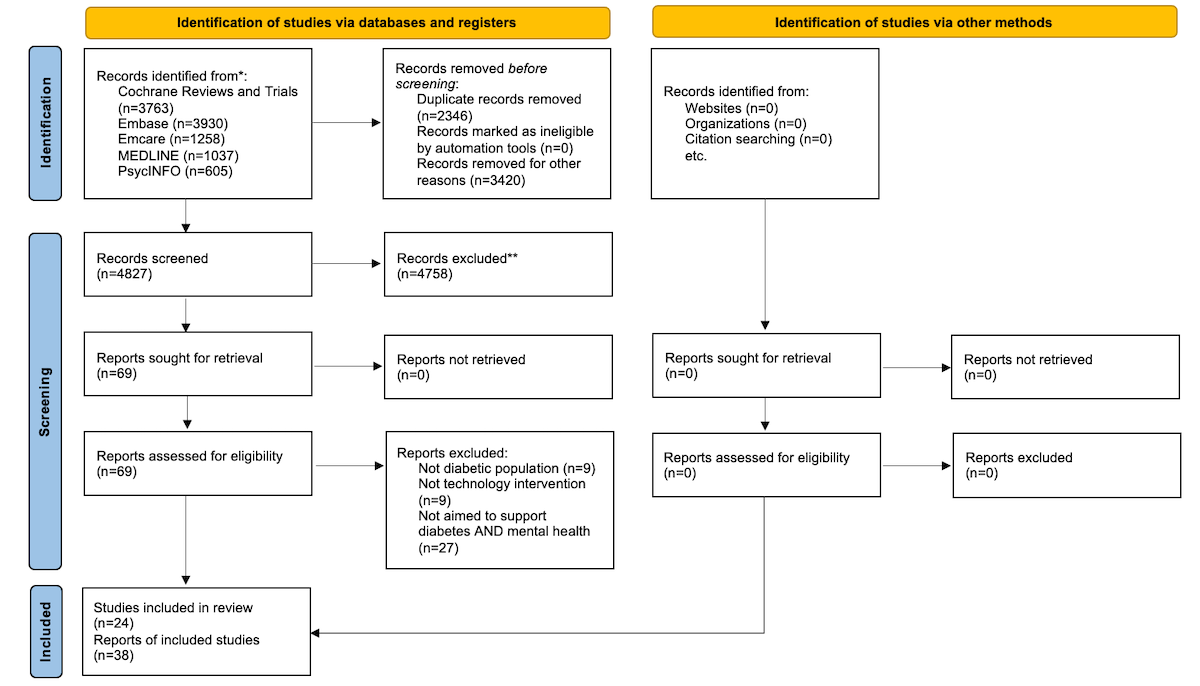
PRISMA (Preferred Reporting Items for Systematic Reviews and Meta-Analyses) diagram. *Consider, if feasible to do so, reporting the number of records identified from each database or register searched (rather than the total number across all databases/registers). **If automation tools were used, indicate how many records were excluded by a human and how many were excluded by automation tools.

Our search yielded 4827 citations after duplicates were removed (see Figure 1). We assessed 69 full-text citations for eligibility and excluded 45 of these studies mostly because they were not aimed to support both diabetes and mental health (either concurrently or in succession). The remaining 24 primary studies [21-44] described in 38 publications [45-58] were included in a qualitative synthesis for this scoping review (Figure 1). The included studies were mostly randomized controlled trials or controlled clinical trials (n=14; Tables 1-3). Other study designs included one-group pre-post studies (n=5) [23,31,32,41,44], systematic or literature reviews (n=3) [22,34,40], mixed methods (n=1) [33], and focus groups (n=1) [21].

**Table 1:** Characteristics of included studies by a methodological approach: quantitative studies.

Author, year (ref)^a^	Study design	Sample size, n	Diabetes type	Focus of study	Length of study	Delivery person(s)	Site of care^b^	Outcomes measured^c^
Alessi et al [25], 2021	RCT^d^	91	T2D^e^	Mental health	16 weeks	Case manager	Clinic	Patient reported
Bakhach et al [28], 2019	CCT^f^	81	T1D^g^	Combined diabetes and mental health	12 months	Medical doctor or nurse practitioner	Clinic	Patient reported
Bendig et al [24], 2021	RCT	42	T1D and T2D	Combined diabetes and mental health	8 weeks	Psychologist	Primary care	Patient reported, process
Bond et al [37], 2010	RCT	62	N/R^h^	Combined diabetes and mental health	6 months	Nurse, social worker, or a PhD psychologist	Web-based	Patient reported
Clarke et al [41], 2016	One group pre-post design	91	T1D and T2D	Mental health	7 weeks	N/A^i^ (self-guided)	Web-based	Patient reported
Clarke et al [27,47,48], 2019	RCT	780	T2D	Mental health	8 weeks	N/A (self-guided)	Web-based	Patient reported, health, process, other (health service use, days out of role)
Cohn et al [43], 2014	RCT	53	T2D	Mental health	5 weeks	N/A (self-guided)	Diabetes clinic	Patient reported, Process
Crawford et al [39], 2019	RCT	88	T1D and T2D	Mental health	3 days	N/A	Web-based	Patient reported, process, other (health care use)
DuBois et al [32], 2016	One group pre-post design	15	T2D	Mental health	12 weeks	Study trainer	Hospital and clinic	Patient reported, process
Magee et al [23], 2021	One group pre-post design	18	T2D	Combined diabetes and mental health	12 weeks	Social worker	Hospital	Patient reported, health, process
Mochari-Greenberger et al [31], 2016	One group pre-post design	466	T1D and T2D	Mental health	8 weeks	Social worker and behavioral coach	Web-based	Patient reported, health
Murray et al [29], 2017	RCT	374	T2D	Combined diabetes and mental health	12 months	Nurse facilitated but self-guided	Clinic	Patient reported, health, process
Naik et al [44], 2012	One group pre-post design	8	T2D	Mental health	12 weeks	Clinical psychologist, a developmental psychology postdoctoral fellow, doctoral student, and psychology student	Telephone	Patient reported, health
Naik et al [26,45,46], 2019	RCT	225	N/R	Combined diabetes and mental health	12 months	Psychologists, nurses, pharmacists, and social workers	MEDVAC clinic and telephone	Patient reported, health, other (health care use)
Newby et al [38,55], 2017	RCT	91	T1D and T2D	Mental health	10 weeks	N/A (self-guided)	Web-based	Patient reported, process
Nobis et al [30,49-51], 2015	RCT	260	T1D and T2D	Combined diabetes and mental health	8 weeks	Coaches	Web-based	Patient reported, health, process, other (cost)
Orman et al [33], 2016	Pre-post mixed method	35	T1D and T2D	Mental health	4 weeks	N/A (self-guided)	Web-based	Patient reported, health, process
Piette et al [35,52], 2011	RCT	291	T2D	Combined diabetes and mental health	12 months	Registered nurse	Telephone	Patient reported, health
van Bastelaar et al [36,53,54], 2011	RCT	255	T1D and T2D	Mental health	N/R	Certified health psychologists	Web-based	Patient reported, health
Wu et al [42,56-58], 2018	CCT	1406	T2D	Mental health	12 months	Nurse care managers, nurse practitioners, physician, and social worker	Clinic	Patient reported, health, process, other (cost)

^a^References for primary papers and any related publications.

^b^Site of care for any intervention components beyond the technology.

^c^Outcome types: “process” includes study feasibility and adherence; “patient reported” includes quality of life, distress, anxiety, depression, and knowledge; “health” includes glucose measures, body composition, and cardiometabolic outcomes; and “other” is as described in the table including cost, provider experience, and health service use.

^d^RCT: randomized controlled trial.

^e^T2D: type 2 diabetes.

^f^CCT: controlled clinical trial.

^g^T1D: type 1 diabetes.

^h^N/R: not reported.

^i^N/A: not applicable.

**Table 2:** Characteristics of included studies by a methodological approach: qualitative studies.

Author, year (ref)^a^	Study design	Sample size, n	Diabetes type	Focus of study	Length of study	Delivery person(s)	Site of care^b^	Outcomes measured
Boggiss et al [21], 2021	Qualitative focus groups	16	T1D^c^	Mental health	3 months	Health psychology PhD candidate and registered health psychologist	Web-based	N/A^d^

^a^References for primary papers and any related publications.

^b^Site of care for any intervention components beyond the technology.

^c^T1D: type 1 diabetes.

^d^N/A: not applicable.

**Table 3:** Characteristics of included studies by a methodological approach: reviews.

Author, year (ref)^a^	Study design	Number of studies	Diabetes type	Aim of review	Inclusion criteria	Exclusion criteria	Main finding	Outcomes measured^b^
Franco et al [40], 2018	Review	5	T1D^c^ and T2D^d^	Describe web-based interventions for depression in individuals with diabetes and to discuss these studies’ procedures and findings in light of evidence from a wider range of interventions for depression and diabetes	Inclusion: published in English or Spanish in a peer-reviewed journal between 1990 and 2017, participants (18 years or older) with a primary diagnosis of diabetes and comorbid depression, program content multimedia; provision of web-based activities; and a guided or unguided self-help approach, target depression symptomatology with the specific intent of producing emotional, behavioral, and cognitive change, studies with repeated measure designs	—^e^	4 studies found a significant reduction in depression scores and diabetes distress in the intervention condition compared with control	Patient reported, health
van der Feltz-Cornelis [34], 2013	Literature Review	N/R^f^	N/R	What treatments of comorbid depression in diabetes mellitus can positively impact diabetes disease control, and what evidence for this view has emerged since 2010, with a focus on psychotherapeutic and pharmacotherapeutic versus eHealth or mHealth^g^ interventions?	N/R	N/R	Face-to-face treatment appears to remain the treatment mode of choice, be it psychotherapy or pharmacotherapy. CBT^h^, as well as pharmacotherapy, is effective in terms of depression outcomes. Results of eHealth and mHealth show that the improvement of glycemic control was small, both in patients with diabetes with and without depression. Interventions specifically aimed at improving glycemic control by eHealth or mHealth only show limited results	Patient reported, health
Yap et al [22], 2021	Systematic review and meta-analysis	20	T2D	Synthesized the best available evidence concerning the effectiveness of TBPIs^i^ on diabetes distress, self-efficacy, HRQoL^j^, and HbA_1c_^k^ level among adults with T2D	At least 18 years old and with the diagnosis of T2D; tested TBPIs (such as motivational interviewing, behavioral therapy, and CBT); compared TBPIs with usual care, enhanced usual care, waiting list, and attentional control groups; measured at least one of these outcomes: DD^l^, self-efficacy, HRQoL or HbA_1c_ levels with validated measuring tools; used randomized controlled trials that were reported in English from 2010 to 2020; interventions delivered by health care providers and comprised more than 50% in-person sessions	Studies with self-help groups, peer-delivered interventions or general education	All outcomes except HRQoL were statistically significant with a small effect size	Patient reported, health

^a^References for primary papers and any related publications.

^b^Outcome types: “patient reported” includes quality of life, distress, anxiety, depression, and knowledge; and “health” includes glucose measures, body composition, and cardiometabolic outcomes.

^c^T1D: type 1 diabetes.

^d^T2D: type 2 diabetes.

^e^Not available.

^f^N/R: not reported.

^g^mHealth: mobile health.

^h^CBT: cognitive behavioral therapy.

^i^TBPI: technology-based psychosocial intervention.

^j^HRQol: health-related quality of life.

^k^HbA_1c_: hemoglobin A_1c_.

^l^DD: diabetes distress.

### Study Characteristics

**Figure 2:**
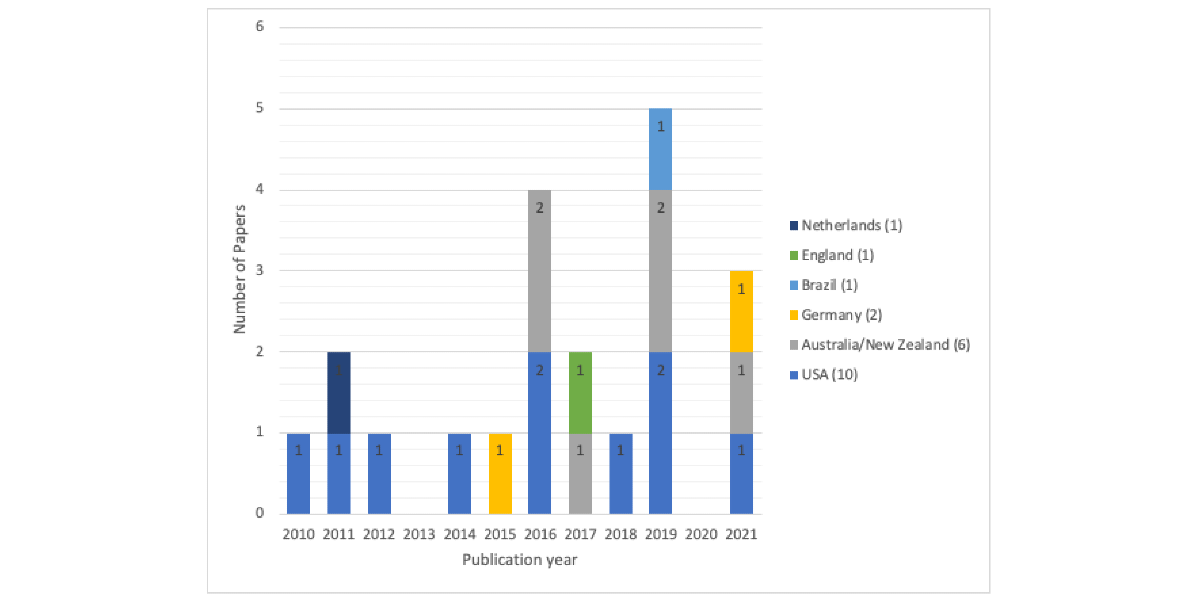
Number of included papers by year and country. Note: 3 reviews are not included in this figure.

The characteristics of the primary papers can be found in Tables 1-3, and the full characteristics of the individual primary papers can be found in Multimedia Appendix 2. A total sample of 4748 adults with diabetes were included in this review. Studies included mostly participants with T2D (n=10) or mixed diabetes populations (n=9). Only 2 studies were conducted with participants with T1D (one qualitative focus group study [21] and the other a controlled clinical trial diabetes and mental health intervention [28] representing a total sample of 97 adults with T1D specifically), whereas in a further 3 studies, the diabetes population was unclear [26,34,37]. None of the included studies specifically reported including participants with GD. The publication years of the included studies suggest an increasing trend as 14 of the studies were published in the last 5 years and most of the publications were within the last 3 years of the review period (Figure 2). The papers originated from the USA (n=10), Australia (n=5), Germany (n=2), and 1 paper each from the following countries: Brazil, England, Netherlands, and New Zealand. Studies were generally of short duration with half the studies (n=12) being ≤12 weeks long, 2 between 13 and 26 weeks in duration, and 5 were 12 months long. Eight studies had a follow-up outcome measurement beyond the end of the study time point. Of the 20 intervention studies, 12 focused on addressing mental health in people living with diabetes [25,27,31-33,36,38,39,41-44] and 8 addressed both diabetes and mental health conditions concurrently [23,24,26,28-30,35,37].

### Technology-Enabled Integrated Interventions for Diabetes and Mental Health

#### Purpose of Use

**Figure 3:**
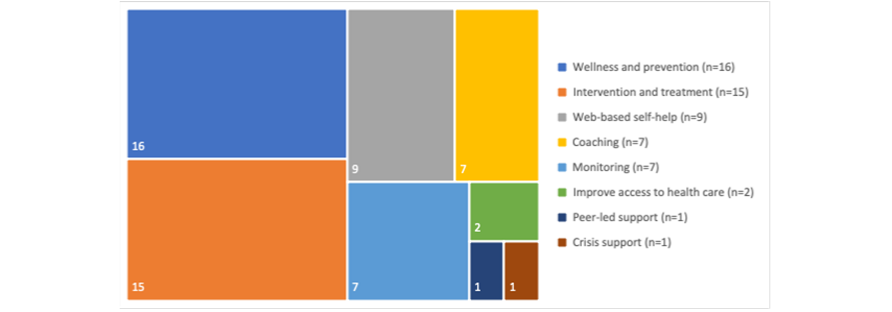
Tree map of the purpose of technology used in included studies as reported in 20 studies. Note: as per the definitions from the Mental Health Commission of Canada “Mental Health, Technology, and You,” 3 systematic reviews and 1 focus group study are not part of the data extraction to construct this figure.

Of the 10 purposes of technology described in the Mental Health Commission of Canada framework, 8 were found in our included studies (Figure 3; Multimedia Appendix 3). The overwhelming majority of the included studies used technology for wellness and prevention (n=16; ie, practicing healthy habits to maintain or improve health) and intervention and treatment (n=15; ie, specialist consultation, assessment, or follow-up). The other 2 most common purposes were web-based self-help (n=9; ie, accessing resources) and coaching (n=7; ie, working with a trained professional). Very few studies used the technology to improve access to health care (n=2) [29,33], to provide peer-led support (n=1) [37], or to provide crisis support (n=1) [42].

#### Context of Use

Studies were conducted in a range of settings and sites of care (Tables 1-3), and some included both web-based and in-person settings. Over half the studies (n=13) were delivered primarily through web-based platforms, websites, apps, or phone calls, and about one-third (n=9) of the studies included primary care settings such as doctor’s offices, hospitals, or clinic visits. A variety of health care professionals and staff were used to help deliver the services and technologies to participants (Tables 1-3). Although 6 studies were entirely automated or self-directed by the participants, with no involvement by any health care professionals or staff, the remaining studies used 1 or more of the following: psychologist (n=6), nurse (n=6), social worker (n=5), graduate student (masters or doctoral level) or postdoctoral fellow (n=3), medical doctor or physician (n=2), and 1 study each for case manager, pharmacist, and behavior coach. Their involvement ranged from supervising, training, and overseeing the implementation of the research project, to directly supporting the implementation of the technology component of the study (phone calls, emails, telemedicine, remote feedback, and support through direct or instant SMS text messaging and video), to leading separate and additional components of the study outside of the technology (facilitating discussion groups and one-on-one meetings). Their involvement varied greatly in terms of structured and scripted feedback compared with more personalized and individualized coaching. Both synchronous and asynchronous deliveries were used.

#### Type of Technology Used in the Interventions

We extracted the details of the technology from the included studies that actively evaluated or tested a digital health technology (n=20 intervention studies). The 3 reviews and 1 focus group study were not included in this extraction of data as the general nature of the reviews and investigative purpose of the focus groups did not lend themselves to such specific categorization. Full data extraction on the components of the digital health technologies of the individual primary papers can be found in Multimedia Appendix 3.

Half of the studies (n=13) used computerized treatments, resources, and mobile apps. These were mostly website-based, accessible from many types of devices, and none of these mobile apps were mobile health. These websites served a variety of purposes and included many different features ranging from educational materials to interactive learning modules and discussion boards (Textbox 1). Telehealth or telemedicine was used in 9 of the included studies, some of which also used computerized treatments or apps. Only 1 study used social media or peer support platforms (MSN Messenger) [37], and 1 study used artificial intelligence [42].

**Website components offering diabetes and mental health from included studies.**

- Resources and educational materials with articles and sites on diabetes and other health-related topics
- Interactive learning modules and topics related to diabetes self-care and management and behavior change
- Tracking of personal health data, physical activity and diet records, and participant logbooks and diaries
- Stories from peers or similar target audience, success stories, and emotional well-being support
- Activities and questionnaires
- Discussion boards and moderated forums, with or without involvement from intervention delivery persons
- Receiving advice, instruction, and action plans
- Setting frequency and type of text messaging and daily reminders
- Frequently asked questions

### Primary Users

Applying the World Health Organization’s “Classification of Digital Health Interventions v1.0” framework, primary users of interventions may be categorized as those specific to clients, health care providers, health systems, or data services. In this review, primary users included clients and health care providers, with none of the included studies describing interventions for health systems or data services.

#### Interventions for Clients

**Figure 4:**
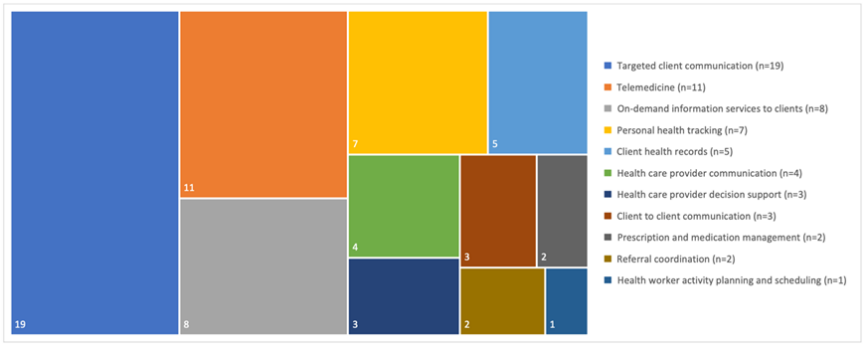
Tree map of the digital health intervention classifications as reported in 20 studies. Note: as per the World Health Organization’s “Classification of Digital Health Interventions v1.0,” 3 systematic reviews and 1 focus group study are not part of the data extraction to construct this figure.

Interventions for clients encompassed 4 of the 7 subgroups (Figure 4): targeted client communication (such as telehealth, phone calls, emails, or SMS text messaging with a provider; n=19), on-demand information services to clients (mostly through access to websites or provision of information through pamphlets and handouts; n=8), personal health tracking (through websites; n=7), and client-to-client communication (such as discussion boards or peer-to-peer messaging systems; n=3) [28,29,37].

#### Interventions for Health Care Providers

Seven of the 10 subgroups were covered by included studies (Figure 4). These were telemedicine (n=11), client health records (n=5) [25,28,31,37,42], health care provider communication (n=4) [25,26,42,44], health care provider decision support (n=3) [35,42,44], referral coordination (n=2) [23,42], prescription and medication management (n=2) [23,42], and health worker activity planning and schedule (n=1) [44]. These intervention components were mostly related to the professional integration of the study.

### Level of Integration of Technology-Enabled Diabetes and Mental Health Support

Included studies only reported interventions operating at 2 of the levels of integration. All the included intervention studies (n=20) used technology for clinical integration, and approximately one-third (n=7) also used the technology for professional integration. Professional integration supports the communication and interaction between colleagues from different disciplines across different organizations [19]. In our included studies, the provision of information and data into the study technology allowed for better coordination, communication, and interaction of health care providers through sharing client records with specialists and other health care professionals, supporting health care decision-making, enabling communication between health care providers, coordination of referrals, and better prescription and medication management. For many studies, professional integration allowed the participants and the delivery persons (including health care providers) to set coordinated clinical care goals and action plans. Wu et al [42] used clinical decision support software as part of their technology intervention. This software automatically generated task reminders and alerts based on the data of patient records that were directed to specific providers.

## Discussion

### Principal Findings

People living with diabetes and mental health challenges may find it difficult to balance self-care demands (eg, maintaining a healthy diet, exercising, or taking medication) [4-6]. Integrated diabetes and mental health care delivery models can help overcome the historically fragmented and siloed care that people living with diabetes and mental health challenges currently must navigate [10]. Health technology, including innovative interventions such as those described in this review, may further drive the scale-up of these integrated solutions. Our review sought to describe technology-enabled integrated interventions for diabetes and mental health and to describe how these interventions are or may be integrated into clinical care.

Our review found that much of the evidence for integrated technology for diabetes and mental health support was derived from clinical controlled trials and pre-post studies. Similar to a previous review by Franco et al [40], we found that our included studies were heterogeneous in terms of whether they focused their intervention to support combined diabetes interventions with mental health components versus mental health–specific interventions in people living with diabetes. This is consistent with prior research trends for both face-to-face and digital health interventions [40]. Moreover, about a quarter of the identified studies did not specifically discuss the mental health intervention components, aims, and goals, and it was unclear what constructs of psychological well-being were targeted (eg, distress, depression, or self-efficacy). This is despite high levels of evidence to suggest that individuals with diabetes value psychoeducation that is illness-specific such as interventions targeting depression [40]. Additional studies that provide a greater detail on the psychological constructs targeted are therefore needed. Similar behavioral treatment strategies and approaches can be used to address both mental health challenges and diabetes, which may contribute to better integration and improved overall health outcomes.

Most of the included studies used web-based technologies, and the majority targeted either mental health wellness and prevention or intervention and treatment support. Several studies identified their purpose as providing web-based self-help and health coaching, and only 2 studies used technology to improve access to health care [29,33]. Specifically, there was evidence of clinical integration (access to diabetes and mental health support); however, only 2 studies described health professional integration across diabetes and mental health care providers [23,42]. This finding highlights the state of integrated care, where clinical integration of diabetes care and mental health support exists but does not quite reach integration of health care professionals at the organization and system level. To that end, of the 7 studies that comprised diabetes and mental health combined interventions, only 3 included professional integration [23,26,35], suggesting that more work needs to be done to prepare health care professionals and organizations for the delivery of integrated care.

Recognizing the impact of COVID-19 and the push for technology-based solutions to deliver care, an abundance of recent papers spoke to the rapid uptake and evolution of technology-based interventions; however, only one of these studies met our inclusion criteria for technology-enabled integrated care [25]. Moreover, the pandemic has highlighted the need for careful consideration as to how technology may further restrict access to care, integration or coordination of care, and ultimately disadvantage some individuals, particularly those who are underserviced or underrepresented in diabetes and mental health care. For example, for those living with mental health challenges, the added time or cognitive investment inherent to some of these interventions may contribute additional burden, disempowerment, and ultimately lead to not seeking help—further reinforcing not only digital inequities but other social and health inequities too [59].

### Future Research

We are consistently moving toward digital systems where interactions and services that were traditionally in-person, by phone, and paper are now only offered via a web platform or virtually. This includes health and social services. In this regard, digital equity is essential for universal access to these services, especially by underserved, marginalized communities. Barriers to digital equity, such as inadequate infrastructure in rural and remote communities, lack of affordable options for high-speed broadband internet, digital literacy, or digital poverty, must be considered as more technology-based health care services emerge. With an emphasis on equity, diversity, and inclusion, future research should carefully consider a staggered but not exclusive approach for offering self-management solutions to integrated clinical health care provider and organizational models of technology-enabled care. Further, the development of these interventions must be done in tandem with measurement-based approaches to digital health equity [59]. In particular with Indigenous populations, integration of mental health and physical health is important culturally in the development of effective health care interventions and services.

### Strengths and Limitations

Our review has several strengths. First, we used a rigorous process to search, screen, and select on-topic literature, following best-practice methodologies [16,60]. Our search is current and includes papers as recent as February 2022 from multiple databases. Finally, we used several theoretical frameworks to dissect key concepts about technology-enabled diabetes and mental health integrated care including frameworks to understand key concepts such as the intervention, the purpose of the technology, and the intent of the integration. Although our review did not critically appraise the literature or perform a meta-analysis, our methods align with the aims and purpose of a scoping review [14,61]. By only including studies written in English, we may have missed important papers written in other languages.

### Conclusions

Despite the growing need for diabetes and mental health support, there remains limited access to interprofessional, integrated care that includes psychological support, education, and follow-up for individuals living with diabetes and their comorbidities (eg, depression, anxiety, or distress). The findings of this scoping review suggest that there is an emergence of literature pertaining to health technology for diabetes and mental health integrated care. Future research is needed to continue to explore the purpose, level, and breadth of technology-enabled integration to facilitate an approach to overcome or address care fragmentation for diabetes and mental health.

## Appendix

Multimedia Appendix 1 Search strategy.

Multimedia Appendix 2 Characteristics of included studies.

Multimedia Appendix 3 Overview of digital health technology.

## Abbreviations

GD: gestational diabetes
PRISMA: Preferred Reporting Items for Systematic Reviews and Meta-Analyses
T1D: type 1 diabetes
T2D: type 2 diabetes

## Data Availability

The main study data are the data extraction materials and quality ratings of included papers, most of which are included in the manuscript tables. Any other supporting data relating to this review are available by request from the authors.
