# Supplementary material for: Technology-Supported Integrated Care Innovations to Support Diabetes and Mental Health Care: Scoping Review
Source: JMIR Diabetes. 2023 May 9;8:e44652. doi: 10.2196/44652 (PMC10206630; doi:10.2196/44652)
Supplement: Multimedia Appendix 1 [file diabetes_v8i1e44652_app1.docx]

**Supplemental Material 1: Search Strategy**

**DIABETES AND MENTAL HEALTH—DIGITAL INTERVENTIONS**

**February 2022**

Database: OVID **Medline** Epub Ahead of Print, In-Process & Other Non-Indexed Citations, Ovid MEDLINE(R) Daily and Ovid MEDLINE(R) 1946 to Present

Search Strategy:

--------------------------------------------------------------------------------

1 exp Diabetes Mellitus/ (468489)

2 diabet*.af. (866384)

3 1 or 2 (868483)

4 exp Mental Disorders/ (1349898)

5 exp Stress, Psychological/ (144949)

6 (depression or ((depressive or mood or bipolar) adj2 (symptom? or disorder?))).af. (547035)

7 (suicide? or suicidal).af. (105043)

8 (anxiety or anxious or distress or ((emotional or psychological or financial or occupational or work or workplace) adj2 (stress or burnout))).af. (524899)

9 ((psychologic* or psychiatric or mental or emotional or mental health) adj2 (disorder? or condition? or problem? or comorbid*)).af. (303853)

10 exp Behavioral Symptoms/ (410713)

11 ((substance? or alcohol or drug? or narcotic? or cocaine or heroin or methamphetamine? or crystal meth or cannabis or marijuana or opioid? or opiate? or prescription drug?) adj2 ("use" or abuse or dependence or withdrawal or disorder? or overdose)).af. (377398)

12 (alcoholic or alcoholism).af. (174570)

13 or/4-12 (2283687)

14 3 and 13 (62095)

15 ((Digital or virtual or remote or computer* or web-based or internet or technology-based or technology-assisted) adj2 (intervention? or program* or therapy or care or application* or monitor*)).af. (76552)

16 exp Telephone/ (32350)

17 Telemedicine/ (32250)

18 internet/ (78318)

19 (smartphone? or smart phone? or cell phone? or phone? or text messag* or app? or sms).af. (117645)

20 Self-Help Devices/ (5385)

21 (electronic mail or email or e-mail).ti,ab. (16319)

22 Electronic Mail/ (2870)

23 or/15-22 (305830)

24 14 and 23 (1060)

25 limit 24 to english language (1037)

Database: **Embase** <1974 to 2022 February 10>

Search Strategy:

--------------------------------------------------------------------------------

1 exp diabetes mellitus/ (1076098)

2 diabet*.af. (1431924)

3 1 or 2 (1436763)

4 exp mental disease/ (2396295)

5 exp mental stress/ (174470)

6 (depression or ((depressive or mood or bipolar) adj2 (symptom? or disorder?))).af. (832087)

7 (suicide? or suicidal).af. (145604)

8 (anxiety or anxious or distress or ((emotional or psychological or financial or occupational or work or workplace) adj2 (stress or burnout))).af. (700959)

9 ((psychologic* or psychiatric or mental or emotional or mental health) adj2 (disorder? or condition? or problem? or comorbid*)).af. (258467)

10 ((substance? or alcohol or drug? or narcotic? or cocaine or heroin or methamphetamine? or crystal meth or cannabis or marijuana or opioid? or opiate? or prescription drug?) adj2 ("use" or abuse or dependence or withdrawal or disorder? or overdose)).af. (864705)

11 (alcoholic or alcoholism).af. (226055)

12 or/4-11 (3585375)

13 3 and 12 (176818)

14 ((Digital or virtual or remote or computer* or web-based or internet or technology-based or technology-assisted) adj2 (intervention? or program* or therapy or care or application* or monitor*)).af. (282268)

15 telemedicine/ or exp teleconsultation/ or telemonitoring/ or telepsychiatry/ or telepsychology/ or teletherapy/ or video consultation/ (51941)

16 telephone/ (41391)

17 internet/ or web-based intervention/ (117641)

18 (smartphone? or smart phone? or cell phone? or phone? or text messag* or app? or sms).af. (182102)

19 exp mobile phone/ (38079)

20 exp mobile application/ (18780)

21 e-mail/ (25956)

22 (electronic mail or email or e-mail).ti,ab. (32315)

23 or/14-22 (647862)

24 13 and 23 (3930)

Database: Ovid **Emcare** <1995 to 2022 Week 6>

Search Strategy:

--------------------------------------------------------------------------------

1 exp diabetes mellitus/ (245790)

2 diabet*.af. (321481)

3 1 or 2 (322431)

4 exp mental disease/ (732291)

5 exp mental stress/ (66531)

6 (depression or ((depressive or mood or bipolar) adj2 (symptom? or disorder?))).af. (250103)

7 (suicide? or suicidal).af. (51455)

8 (anxiety or anxious or distress or ((emotional or psychological or financial or occupational or work or workplace) adj2 (stress or burnout))).af. (250621)

9 ((psychologic* or psychiatric or mental or emotional or mental health) adj2 (disorder? or condition? or problem? or comorbid*)).af. (91380)

10 ((substance? or alcohol or drug? or narcotic? or cocaine or heroin or methamphetamine? or crystal meth or cannabis or marijuana or opioid? or opiate? or prescription drug?) adj2 ("use" or abuse or dependence or withdrawal or disorder? or overdose)).af. (266284)

11 (alcoholic or alcoholism).af. (50301)

12 or/4-11 (1087024)

13 3 and 12 (48526)

14 ((Digital or virtual or remote or computer* or web-based or internet or technology-based or technology-assisted) adj2 (intervention? or program* or therapy or care or application* or monitor*)).af. (79143)

15 telemedicine/ or exp teleconsultation/ or telemonitoring/ or telepsychiatry/ or telepsychology/ or teletherapy/ or video consultation/ (19644)

16 telephone/ (23417)

17 internet/ or web-based intervention/ (54829)

18 (smartphone? or smart phone? or cell phone? or phone? or text messag* or app? or sms).af. (64513)

19 exp mobile phone/ (11938)

20 exp mobile application/ (6753)

21 e-mail/ (11630)

22 (electronic mail or email or e-mail).ti,ab. (12256)

23 or/14-22 (229524)

24 13 and 23 (1258)

Database: APA **PsycInfo** <1806 to January Week 5 2022>

Search Strategy:

--------------------------------------------------------------------------------

1 exp diabetes mellitus/ (9408)

2 diabet*.ti,ab. (33811)

3 1 or 2 (34461)

4 exp mental disorders/ (920639)

5 exp anxiety management/ (1007)

6 exp anxiety/ (81215)

7 atypical depression/ (211)

8 "depression (emotion)"/ (26417)

9 exp stress/ (128092)

10 (depression or ((depressive or mood or bipolar) adj2 (symptom? or disorder?))).af. (818122)

11 (suicide? or suicidal).af. (185365)

12 (anxiety or anxious or distress or ((emotional or psychological or financial or occupational or work or workplace) adj2 (stress or burnout))).af. (792877)

13 ((psychologic* or psychiatric or mental or emotional or mental health) adj2 (disorder? or condition? or problem? or comorbid*)).af. (633032)

14 psychiatric symptoms/ (17190)

15 ((substance? or alcohol or drug? or narcotic? or cocaine or heroin or methamphetamine? or crystal meth or cannabis or marijuana or opioid? or opiate? or prescription drug?) adj2 ("use" or abuse or dependence or withdrawal or disorder? or overdose)).af. (450431)

16 (alcoholic or alcoholism).af. (143807)

17 or/4-16 (1934586)

18 3 and 17 (17857)

19 exp telemedicine/ (10696)

20 ((Digital or virtual or remote or computer* or web-based or internet or technology-based or technology-assisted) adj2 (intervention? or program* or therapy or care or application* or monitor*)).af. (74492)

21 exp mobile devices/ (9621)

22 computer mediated communication/ (6275)

23 exp electronic communication/ (38878)

24 internet/ (30121)

25 (electronic mail or email or e-mail).ti,ab. (8705)

26 (smartphone? or smart phone? or cell phone? or phone? or text messag* or app? or sms).ti,ab. (28751)

27 or/19-26 (162499)

28 18 and 27 (676)

29 limit 28 to human (605)

**Cochrane**

Search Name: DM and mental health--digital care

Date Run: 08/02/2022 07:59:15

Comment:

ID Search Hits

#1 MeSH descriptor: [Stress, Psychological] explode all trees 6674

#2 MeSH descriptor: [Mental Disorders] explode all trees 79322

#3 (depression or ((depressive or mood or bipolar) near/2 (symptom? or disorder?))) 95695

#4 suicide? or suicidal 7348

#5 (anxiety or anxious or distress or ((emotional or psychological or financial or occupational or work or workplace) near/2 (stress or burnout))) 86502

#6 ((psychologic* or psychiatric or mental or emotional or mental health) near/2 (disorder? or condition? or problem? or comorbid*)) 44562

#7 MeSH descriptor: [Behavioral Symptoms] explode all trees 24130

#8 ((substance? or alcohol or drug? or narcotic? or cocaine or heroin or methamphetamine? or crystal meth or cannabis or marijuana or opioid? or opiate? or prescription drug?) near/2 ("use" or abuse or dependence or withdrawal or disorder? or overdose)) 170468

#9 alcoholic or alcoholism 14485

#10 #1 or #2 or #3 or #4 or #5 or #6 or #7 or #8 or #9 354772

#11 ((Digital or virtual or remote or computer* or web-based or internet or technology-based or technology-assisted) near/2 (intervention? or program* or therapy or care or application* or monitor*)) 16289

#12 MeSH descriptor: [Telephone] explode all trees 4361

#13 MeSH descriptor: [Telemedicine] explode all trees 3119

#14 MeSH descriptor: [Internet] explode all trees 4545

#15 (smartphone? or smart phone? or cell phone? or phone? or text messag* or app? or sms) 43783

#16 MeSH descriptor: [Self-Help Devices] explode all trees 435

#17 (electronic mail or email or e-mail):ti,ab,kw 5229

#18 MeSH descriptor: [Electronic Mail] explode all trees 354

#19 #12 or #13 or # 14 or #15 or #16 or #17 or #18 296583

#20 (diabet*):ti,ab,kw 103189

#21 MeSH descriptor: [Diabetes Mellitus] explode all trees 34217

#22 #20 or #21 103461

#23 #10 and #19 and #22 3794

#24 #10 and #19 and #22 in Cochrane Reviews 297

#25 #10 and #19 and #22 in Trials 3466
