# Supplementary material for: Technology-Supported Integrated Care Innovations to Support Diabetes and Mental Health Care: Scoping Review
Source: JMIR Diabetes. 2023 May 9;8:e44652. doi: 10.2196/44652 (PMC10206630; doi:10.2196/44652)
Supplement: Multimedia Appendix 2 [file diabetes_v8i1e44652_app2.docx]

**Supplemental Material 2: Characteristics of Included Studies (COIS)**

| Author, Year | Alessi, 2021 |
| --- | --- |
| Aim of study | Aimed to assess the impact of teleinterventions on mental health parameters in those with type 2 diabetes during the COVID-19 pandemic in Brazil. |
| Sample size | 91 |
| Population: Inclusion/exclusion criteria | Inclusion: ≥18 years with a previous diagnosis of T2D based on guideline recommendations; HbA1c evaluation in the laboratory of the study’s reference hospital in the three months prior to inclusion; and availability for weekly phone calls during the study  Exclusion: hospitalized at the time of recruitment and those who had some serious limitation preventing the necessary interaction, such as advanced dementia or severe hearing loss |
| Population: Diabetes type | T2D |
| Population: Diabetes diagnosis duration (years, months) | N/R |
| Population: mental health disorder (if applicable) | N/A |
| Population: Mean age (year +/- SD) | 61.3 (9.1) |
| Population: Sex (N male and %) | 32(35) |
| Study design | Open-label randomised controlled trial |
| Methods of study | At randomization, the active control group received access to a website prepared by the multidisciplinary team. On this site, there were weekly posts about diabetes care, mental health, and lifestyle habits, similar to phone call protocols. This group did not have direct contact with the researchers during 16 weeks of the study.  Telehealth intervention: Each participant was randomly assigned to be followed by a same trained researcher, called case manager, responsible for making weekly calls for 16 weeks of follow-up. The calls lasted around 5-10 min and followed a pre-established script to ensure all patients received a similar intervention. Intervention protocols were developed by physicians, physical educators, and psychologists. Each week, a different topic was selected for participants. Topics included issues related to mental health and coping strategies, physical activity, healthy eating habits, and diabetes care.  Educational materials: Patients with difficulty in a specific area related to mental health or diabetes care received additional digital educational material. Information about healthy eating habits and physical exercise adapted to age and physical limitations were offered to all participants.  Clinical care: Intervention sought to complement the usual diabetes care. Adherence to treatment was encouraged on every call. Patients were routinely asked for reports on glycemic controls, following their provider’s recommendation. Treatment adjustments were made in severe cases, such as recurrent hypoglycemia and difficulty in contacting health care providers. In those cases, treatment was recommended by an endocrinologist from the study team. |
| Delivery person(s) | Case manager (trained researcher; not reported whether health professional) |
| Study length AND  Start and end date of study | Study length: 16 weeks study conducted April 2020 - August 2020  recruitment April 2020 |
| Study outcome timepoints | Baseline; 16 weeks post intervention |
| Outcome types -> Patient reported | QoL, distress, anxiety, depression, knowledge, etc |
| List Outcomes and Measurement tool | Mental health outcome: Self Report Questionnaire- 20 (SRQ-20)  Diabetes-related emotional distress: Brazilian validated version of PAID Scale (B-PAID) Sleep disorder: Brazilian version of the MSQ  Eating disorder: Brazilian validated version of EAT–26 Treatment adherence: Brazilian-Portuguese version of the SCIR |
| Location (city, country) | Brazil (southern) |
| Healthcare setting and context/site of care | Out-patient follow-up; community-based |

| Author, Year | Bakhach, 2019 |
| --- | --- |
| Aim of study | Evaluated the impact of participation in the CoYoT1 Clinic on psychosocial and behavioral outcomes related to diabetes, including diabetes distress, self-efficacy, self management, and depressive symptoms, compared to patients receiving usual care. |
| Sample size | 81 |
| Population: Inclusion/exclusion criteria | Inclusion: 18-25 years of age being seen for T1D at the BDC; internet-connected device; physically located in the state of Colorado.  Exclusion: N/R |
| Population: Diabetes type | T1D |
| Population: Diabetes diagnosis duration (years, months) | INT: 8.2 (4.7); CON: 11.7 (5.5) |
| Population: mental health disorder (if applicable) | N/A |
| Population: Mean age (year +/- SD) | INT: 19.8 (1.7); CON: 20.5 (1.9) |
| Population: Sex (N male and %) | INT: 19 (45); CON: 19 (41) |
| Study design | prospective cohort pilot study / CCT (Controlled clinical trial) |
| Methods of study | The CoYoT1 Clinic is a combined SMA-telemedicine intervention in which YAs participate in an individual telemedicine appointment with a diabetes provider (MD or nurse practitioner) and a group telemedicine session with other YAs with T1D, which is facilitated by a certified diabetes educator. Participants completed visits every 3 months over a 12-month time period. Group and individual telemedicine sessions were completed at visits 1, 2 and 4 online. Visit 3 was completed in person to allow for an annual physical examination. No group visit was completed for the in-person appointment. Participants were able to connect virtually to their appointment from any location of their choosing, using an Internet-connected device equipped with a camera and speakers. Before appointments, YAs were instructed to upload data reports from their diabetes management devices. Four to 6 participants were scheduled for each ~30-minute group appointment. Discussions started with an introductory icebreaker activity led by a facilitator. The session then moved to a patient-driven learning and discussion format focusing on topics relevant to YAs with T1D. Group session topics included personal introductions and review of each YA’s “diabetes story,” discussions about diabetes in a school and/or workplace setting, keys to developing self-advocacy and self-efficacy in diabetes, stress management, diabetes burnout, use of diabetes technology to improve quality of life, and transitioning to adult care. |
| Delivery person(s) | MD or nurse practitioner |
| Study length AND  Start and end date of study | Study length: 12 months N/R |
| Study outcome timepoints | Baseline; 12 months post intervention |
| Outcome types -> Patient reported | QoL, distress, anxiety, depression, knowledge, etc |
| List Outcomes and Measurement tool | Diabetes distress: Diabetes Distress Scale Self-efficacy: Self-Efficacy for Diabetes Scale Self-management: Self-Management of Type 1 Diabetes in Adolescence  Depressive symptoms: Center for Epidemiologic Studies Depression Scale (CES-D) |
| Location (city, country) | Colorado, USA |
| Healthcare setting and context/site of care | Clinic |

| Author, Year | Bendig, 2021 |
| --- | --- |
| Aim of study | Evaluated the feasibility of the programme ACTonDiabetes: ACTonDiabetes was built on the ACTonPain treatment programme to develop a widely practicable, local and temporal flexible, low-threshold internet intervention to reduce diabetes distress in people with type 1 and type 2 diabetes. |
| Sample size | 42 |
| Population: Inclusion/exclusion criteria | Inclusion: ≥18 year; self-reported diagnosis of type 1 or type 2 diabetes; internet access; sufficient German language skills and provided written informed consent  Exclusion: other type of diabetes (gestational) |
| Population: Diabetes type | Mixed: T1D, T2D |
| Population: Diabetes diagnosis duration (years, months) | N/R |
| Population: mental health disorder (if applicable) | N/A |
| Population: Mean age (year +/- SD) | 46.9 (14.4) |
| Population: Sex (N male and %) | 21 (50) |
| Study design | feasibility parallel group, randomised controlled trial |
| Methods of study | Theory: ACT The guided internet intervention was delivered on the online platform Minddistrict. Contents were adapted to the population of people with diabetes from the already evaluated ACTonPain intervention. The intervention consists of seven modules. The content of the intervention involves psychoeducation underpinned by fictional peer support (a woman with recently diagnosed T2D, a younger man with T1D, an older man with long-standing, insulin-treated T2D). The narratives of the peers address typical problems and burdens, worries, needs and experiences with strategies and resources as well as topics covered in the intervention. Contents are provided by means of texts, video sequences, audio files or illustrations of various ACT models and metaphors.  Participants were advised to process through the intervention in 7 weeks, completing one module per week. The processing time for each module was scheduled to take approximately 45–60 min. ACTonDiabetes is based on three assumed effect factors: the intervention contents, guidance and daily exercises. Guidance was provided by an e-coach (psychologist) who provided standardised feedback after each module. To support transfer into everyday life, specific ACT-based exercises and tasks (eg, keeping a DD diary) were included. Furthermore, participants received SMS coaching with motivational prompts and mindfulness tasks. |
| Delivery person(s) | Psychologist (e-coach) |
| Study length AND  Start and end date of study | Study length: 7 weeks Enrollment: October 2017 - April 2018 |
| Study outcome timepoints | Baseline; 8 weeks post intervention |
| Outcome types -> Process | study feasibility, adherence, etc |
| Outcome types -> Patient reported | QoL, distress, anxiety, depression, knowledge, etc |
| List Outcomes and Measurement tool | Trial recruitment and acceptability Access routes: open-ended question asking how participants got to know about the study Acceptability: Attitudes towards Psychological Online Interventions Questionnaire (APOI), usage, and engagement of online platform Formative user feedback: voluntary feedback questionnaire after each module Negative effects: Inventory for the Assessment of Negative Effects of Psychotherapy [INEP] Treatment satisfaction: Client Satisfaction Questionnaire (CSQ-8) Adherence: study attrition at 8 weeks post-randomization  Diabetes distress: PAID Depression: PHQ-9 Anxiety: GAD-7 Self-management: DSMQ Diabetes acceptance: AADQ Quality of life (AQoL-8D) Fear of progression: FoP-Q-SF |
| Location (city, country) | Germany |
| Healthcare setting and context/site of care | Varied community/primary care; community-based |

| Author, Year | Boggiss, 2021 |
| --- | --- |
| Aim of study | Examined the perceptions of digital mental health interventions among adolescents with T1D in order to better inform researchers, health professionals, and digital platform developers in general. |
| Sample size | 16 |
| Population: Inclusion/exclusion criteria | Inclusion: 12-16 years adolescent population in Auckland's paediatric clinics; diagnosis of T1D for more than 6 months; no diagnoses of serious developmental or psychiatric disorders; no untreated hypothyroidism or severe hypoglycaemia and English speaking  Exclusion: N/R |
| Population: Diabetes type | T1D |
| Population: Diabetes diagnosis duration (years, months) | N/R |
| Population: mental health disorder (if applicable) | N/A |
| Population: Mean age (year +/- SD) | 14.8 (1.4) |
| Population: Sex (N male and %) | 5 (31) |
| Study design | Qualitative focus groups |
| Methods of study | Each focus group was facilitated by a female health psychology PhD candidate, and a registered health psychologist having experience in facilitating group sessions with teenagers with T1D and all facilitators having experience in conducting focus groups. Focus groups were conducted online with adolescents with T1D using Zoom video conferencing. A total of four focus groups were conducted, with one focus group reserved solely for Samoan participants to ensure any cultural considerations were attended to. This group was co-facilitated by a New Zealand/Samoan registered health psychologist. Each focus group followed a semi-structured interview schedule devised by the study team. For example, ‘what do you like about using a [platform] like this one?’, ‘what do you dislike about it?’, ‘what features do you think are important if we develop a [platform] like this?’. Examples of existing efficacious digital mental health programs for youth were shown as prompts to discuss features they liked and disliked, including a variety of apps, chatbots and websites. The Zoom chat function was offered but was not used by any of the participants. Data saturation was discussed after each focus group. |
| Delivery person(s) | Female health psychology PhD candidate, and a registered health psychologist |
| Study length AND  Start and end date of study | Recruitment started on May 21, 2020 and was completed by July 15, 2020. Focus groups were conducted between June 20, 2020 and July 21, 2020. |
| Study outcome timepoints | N/A - qualitative study |
| Outcome types -> Other | cost, provider experience, etc |
| List Outcomes and Measurement tool | N/A - qualitative study |
| Location (city, country) | Auckland, New Zealand |
| Healthcare setting and context/site of care | Zoom - virtual |

| Author, Year | Bond, 2010 |
| --- | --- |
| Aim of study | Investigated the impact of a 6-month Web-based intervention on the psychosocial well-being of older adults with diabetes. |
| Sample size | 62 |
| Population: Inclusion/exclusion criteria | Inclusion: ≥60 years; diagnosis of diabetes for at least 1 year; living independently in the community; and oral fluency in English.  Exclusion: moderate or severe cognitive, visual, or physical impairment or the presence of severe comorbid disease (end-stage renal disease, blindness, terminal illness). No prior computer experience was required. Individuals were eligible regardless of A1C level. |
| Population: Diabetes type | N/R |
| Population: Diabetes diagnosis duration (years, months) | Mean years with diabetes, INT: 17.8 (11.7); CON: 16.1 (10.5) |
| Population: mental health disorder (if applicable) | N/A |
| Population: Mean age (year +/- SD) | INT: 68.2 (6.2); CON: 66.2 (5.7) |
| Population: Sex (N male and %) | INT: 18 (58); CON: 16 (52) |
| Study design | Randomised controlled trial. |
| Methods of study | The intervention delivered via the Internet, and it emphasized the patient’s role in maintaining health and the importance of setting goals and using problem-solving skills to overcome barriers. The primary objective was to improve the participants’ diabetes self-management behaviors and psychosocial wellbeing. Intervention strategies included using behavioral and motivational strategies, using cues to modify perceptions of self-efficacy, and using cues to modify personal beliefs regarding the subject’s ability to affect the progress of the disease and change his or her personal behavior. The intervention served as an adjunct to the usual care provided by the subject’s provider. The primary care physicians of the intervention patients retained full responsibility and control over the patients’ care. Additional strategies included instruction in disease management, diet, and exercise and the introduction of interventions to deal with the physical and emotional demands of the disease. The interaction between the study nurse and the intervention participants occurred using both synchronous communication (instant messaging and chat) and asynchronous communication (e-mail and a bulletin board). In addition, participants accessed a study Web site to enter their blood glucose readings, exercise programs, weight changes, blood pressure, and medication data. The study nurse accessed participants’ logs to monitor changes in their self- management patterns. As part of the intervention, the study nurse contacted the participant via e-mail or through instant messenger and/or chat when there were changes in blood glucose patterns that needed problem solving to resolve. The weekly online educational discussion group treatment component served 2 purposes: to provide educational material and to promote peer support and social interaction through sharing of each person’s personal experiences in dealing with their diabetes. The formal weekly discussion group was delivered by the principal investigator through a weekly online communication forum using MSN Messenger software. The sessions related to depression, burnout, and coping were led by either an MSN social worker or a PhD psychologist. In addition to the weekly formal discussion sessions, the participants had access to one another through e-mail and instant messaging.   Control/Usual Care Group Received standard diabetes care from their provider. No educational or training materials associated with the intervention were provided to participants in the control group. Access to educational materials/classes through traditional face-to-face classroom methods furnished by their health provider and/or via the Internet. |
| Delivery person(s) | Nurse, and discussion groups with social worker or a PhD psychologist. |
| Study length AND  Start and end date of study | Study length: 6 months Phase 1: Sept 2004-Sept 2005; Phase 2: Feb 2005-Feb 2006 |
| Study outcome timepoints | Baseline; 6 months post intervention |
| Outcome types -> Patient reported | QoL, distress, anxiety, depression, knowledge, etc |
| List Outcomes and Measurement tool | Depression: CES-D QoL: PAID  The perception of diabetes-related social support: Diabetes Support Scale Self-efficacy: Diabetes Empowerment Scale (DES) |
| Location (city, country) | Seattle, Washington, USA |
| Healthcare setting and context/site of care | N/R  Online, community |

| Author, Year | Clarke, 2016 |
| --- | --- |
| Aim of study | Assessed initial acceptability and effectiveness for a larger randomized controlled trial of myCompass as an intervention for depression in diabetes. |
| Sample size | 91 |
| Population: Inclusion/exclusion criteria | Inclusion: diagnosed with T1D or T2D diabetes by a GP or endocrinologist; Australian resident aged 18-75 years; access to the Internet via mobile phone and computer; valid email address; symptoms of at least mild depression (score > 4 on the PHQ-9; no previous experience with myCompass.  Exclusion: individuals who endorsed psychotic symptoms on the PSQ |
| Population: Diabetes type | Mixed (T1D, T2D) |
| Population: Diabetes diagnosis duration (years, months) | N/R |
| Population: mental health disorder (if applicable) | Mild depression or more (>4 on PHQ-9) |
| Population: Mean age (year +/- SD) | T1D: 39.91 (11.72)  T2D: 52.78 (10.21) |
| Population: Sex (N male and %) | T1D: 8 (24) T2D: 19 (35) |
| Study design | Within subject, pre-post design (one group, pre/post) |
| Methods of study | The program assessed user symptoms and provided a personalized intervention that facilitated round-the-clock self-monitoring of moods and behaviors (via mobile phone, tablet, or computer) and provided interactive evidence-based learning modules (via tablet and computer). Each module contains 3, 5- to 10-minute sessions, each with an assigned homework task. Users were encouraged to complete 1 module session per week, with the aim of completing 2 full modules during the intervention period. 7 week intervention, and encouraged to use it ad libitum during this time. Users could schedule text messaging (short message service, SMS) or email reminders to facilitate self-monitoring; receive and print graphical feedback about their self-monitoring (including contextual information) on their mobile phone or computer (to monitor change and assist identification of triggers); and elect to receive helpful facts, mental health care tips or motivational statements by SMS text messaging or email. |
| Delivery person(s) | N/A |
| Study length AND  Start and end date of study | Study length: 7 weeks Recruitment March 2013 - November 2013 |
| Study outcome timepoints | Baseline; 7 weeks post intervention; 20 weeks follow-up |
| Outcome types -> Process | Study feasibility, adherence, etc |
| Outcome types -> Patient reported | QoL, distress, anxiety, depression, knowledge, etc |
| List Outcomes and Measurement tool | Depressive symptoms: PHQ-9 Anxiety symptoms: GAD-7 Mental health self-efficacy: MHSES Impact of mental health problems: WSAS Emotional adjustment to diabetes: PAID  Diabetes self-efficacy: DSES Diabetes self-management: SDSCA  Glycemic control - report on recent symptoms of hypoglycemia (headaches, light-headedness, weakness) and hyperglycemia (increased thirst, dry mouth, decreased appetite)  User satisfaction: post intervention questionnaire Experience: qualitative interviews with 18 participants |
| Location (city, country) | Australia |
| Healthcare setting and context/site of care | Online, Community |

| Author, Year | Clarke, 2019 |
| --- | --- |
| Aim of study | Evaluated the Web-based CBT program, myCompass, for improving social and occupational functioning in adults with T2DM and mild-to-moderate depressive symptoms. The impact of treatment on depressive symptoms, DD, anxiety symptoms, and self-care behavior was also examined. |
| Sample size | 780 |
| Population: Inclusion/exclusion criteria | Inclusion: 18 to 75 years; diagnosed with T2DM by a physician; screened positive for depression on the self-report 2-item PHQ-2 (i.e. ≥2); access to an internet-enabled device (eg, computer, tablet, and/or mobile phone). People who screened positive for depression completed the 9-item PHQ (PHQ-9) at screening so that the level of symptom severity could be determined.  Exclusion: insufficient English literacy, extremely severe depressive symptoms on the full PHQ-9 (score >19); probable psychosis (measured by the psychosis screener developed for the Australian National Mental Health and Wellbeing Survey); currently receiving face-to-face counseling or therapy for depression; change in antidepressant medication within the previous 2 months; high suicide risk (assessed by the PHQ-9 Item-9); previous use of the myCompass program. |
| Population: Diabetes type | T2D |
| Population: Diabetes diagnosis duration (years, months) | Age at diagnosis, mean (SD): 47 (10.84) |
| Population: mental health disorder (if applicable) | Depression |
| Population: Mean age (year +/- SD) | 58 (10.35) |
| Population: Sex (N male and %) | 282 (36) |
| Study design | Randomised controlled trial |
| Methods of study | Theory: CBT The core myCompass program consisted of 12 interactive CBT modules and 20 cognitive and/or behavioral variables for self-monitoring. For maximum outcomes, users were recommended to complete 3 modules and 3 self-monitoring variables. The myCompass program offered flexibility for users to select their own CBT modules and self-monitoring variables, or, if they prefer, algorithm-based guidance. It provided access to a range of other resources including SMS text messaging and/or email self-monitoring reminders, home practice activities to facilitate skill generalization, mental health care tips and motivational statements delivered by email/SMS text messaging, and graphical reporting of self-monitoring data. Participants randomized to the myCompass arm were provided access to the full program for 8 weeks. The program recommended that users complete 3 CBT modules and self-monitor up to 3 symptoms or behaviors. A 4-week tailing-off period followed, in which only the symptom monitoring function was accessible. myCompass users received automated and personalized feedback via email about their use of the program’s self-monitoring and module functions in weeks 1, 3, 5, and 7.   Placebo Intervention (Healthy Lifestyles): The placebo control program, Healthy Lifestyles, was adapted from a control program used in previous studies by members of the research team to replicate the mode of delivery and key functionality of myCompass, but without the therapeutic content. The program contains 12 modules that deliver health and lifestyle information across a range of topics (eg, skin care and mobile phone hygiene), interactive exercises, and the potential for program tailoring via a brief survey completed at registration. Program users received an email at weeks 1, 3, 5, and 7, containing a brief reminder to log into the program but no feedback about their program use. They also received a weekly SMS text message containing a fact relevant to the content of Healthy Lifestyles for the first 4 weeks of the intervention period to match the SMS functionality of myCompass. The Healthy Lifestyles program was designed to have high face validity as a health and lifestyle intervention without any symptomatic benefit. Participants had full access to the Healthy Lifestyles program for 8 weeks. |
| Delivery person(s) | N/A - self-guided |
| Study length AND  Start and end date of study | Study length: 8 weeks Recruitment began in September 2015 and continued until November 2017. |
| Study outcome timepoints | Baseline; 3, 6, and 12 months |
| Outcome types -> Process | study feasibility, adherence, etc |
| Outcome types -> Patient reported | QoL, distress, anxiety, depression, knowledge, etc |
| Outcome types -> Health | glucose measures, body comp, cardio, etc |
| Outcome types -> Other | cost, provider experience, etc |
| Outcome types -> Other (COMMENT) | health service utilization, days out of role |
| List Outcomes and Measurement tool | Difference in work and social functioning: WSAS Depression: PHQ-9 Anxiety: GAD-7 DD: 17-item DDS Diabetes self-care: subset of items from SMP-T2D HbA1c Health service utilization for physical and mental health problems  Days out of role Program engagement data: frequency of log-in, number of modules started and completed, and self-monitoring frequency (myCompass only) |
| Location (city, country) | New South Wales and Victoria, Australia |
| Healthcare setting and context/site of care | recruitment from primary care, but intervention was self-guided and online |

| Author, Year | Cohn, 2014 |
| --- | --- |
| Aim of study | Established efficacy relative to an emotion-reporting control group while gathering participant feedback and data on retention in intervention. |
| Sample size | 53 |
| Population: Inclusion/exclusion criteria | N/R |
| Population: Diabetes type | T2D |
| Population: Diabetes diagnosis duration (years, months) | N/R |
| Population: mental health disorder (if applicable) | Diabetes related stress |
| Population: Mean age (year +/- SD) | 54 (SD N/R) |
| Population: Sex (N male and %) | 26 (49) |
| Study design | Randomized controlled trial. |
| Methods of study | Theory: revised Stress and Coping Theory and the Broaden-and-Build Theory of positive emotion. On the first 1–2 days of each week, participants read a brief lesson introducing that week’s skill(s). For the rest of the week, they received a “home practice” assignment consisting of one or more simple practices, such as noticing positive events or tracking progress toward an attainable goal. Participants were asked to visit the website every day to record their home practice and complete the daily emotion reporting questionnaire. New lessons became available 7 days after beginning the previous lesson, provided that the participant completed the home practice at least once. Participants were also allowed to postpone beginning a new lesson if they did not have time to read the material on the day it became available. The intervention targets improvements in depression rather than diabetes-specific cognitions and health behaviors. Participants were told that they can choose to apply the skills to coping with diabetes, or to other domains of life in which they would like to increase positive emotion or cope more effectively. |
| Delivery person(s) | N/R |
| Study length AND  Start and end date of study | Study length: 5 weeks N/R |
| Study outcome timepoints | Baseline; 1 week after final lesson (on average, 57 days from baseline for intervention group); 7 days after timepoint 2 |
| Outcome types -> Process | Study feasibility, adherence, etc |
| Outcome types -> Patient reported | QoL, distress, anxiety, depression, knowledge, etc |
| List Outcomes and Measurement tool | Depressive symptoms: CES-D Perceived stress: Perceived stress scale Positive and negative affect: Differential Emotions Scale Diabetes self-efficacy: Confidence in Diabetes Self-Care DD: DDS Health behaviours: whether they had tested blood sugar (not at all, once, or more than once); taken medication (none, some, all); walking and exercise (<10min, 10-30min, >30min) Study experience: telephone interview |
| Location (city, country) | San Francisco, USA |
| Healthcare setting and context/site of care | Community-based diabetes clinic. |

| Author, Year | Crawford, 2019 |
| --- | --- |
| Aim of study | Evaluated the efficacy of a web-based BFW intervention for adults with T1DM or T2DM (compared to a CW condition) for reducing diabetes distress and increasing benefit finding in diabetes. The secondary outcomes examined were self-rated depression and anxiety symptoms, diabetes self-care, health, and health care utilization. |
| Sample size | 88 |
| Population: Inclusion/exclusion criteria | Inclusion: Consent to participate; age ≥ 18 years; living in Australia; T1D or T2D, self-reported as diagnosed by a general practitioner or endocrinologist; email address and access to the internet; ability to read and write in English with ease  Exclusion: PHQ-9 score >8 or GAD-7 score >8; current suicidal thoughts, as indicated by a response of >1 to item 9 on the PHQ-9 scale; self-reported diagnosis of schizophrenia, bipolar disorder, or a psychotic disorder; self-reported diagnosis of dementia or another cognitive disorder; engagement in current psychological therapy |
| Population: Diabetes type | Mixed (T1D, T2D) |
| Population: Diabetes diagnosis duration (years, months) | N/R |
| Population: mental health disorder (if applicable) | N/A |
| Population: Mean age (year +/- SD) | 53.79 (15.93) |
| Population: Sex (N male and %) | 15 (21) |
| Study design | Randomized controlled trial. |
| Methods of study | Both the BFW and CW conditions involved participants in three 15-minute online writing sessions (once per day for 3 consecutive days), according to the instructions provided. Participants from both conditions continued to receive usual care from their health services. Participants in the BFW condition were asked to write about any positive thoughts and feelings that they had had about their experiences with diabetes. Participants in the CW condition were asked to write in detail about how their time was spent that day (first writing session) and the plans for how their time will be spent the following day (second writing session) and week (third writing session). Participants were instructed to be as objective as possible and to focus on the facts and details of how their time was spent (or will be spent), and not to focus on their emotions. |
| Delivery person(s) | N/A |
| Study length AND  Start and end date of study | Study length: 3 days  Ad/recruitment February 2015 - November 2016 |
| Study outcome timepoints | Baseline; 1 month; 3 months |
| Outcome types -> Process | Study feasibility, adherence, etc |
| Outcome types -> Patient reported | QoL, distress, anxiety, depression, knowledge, etc |
| Outcome types -> Other | Cost, provider experience, etc |
| Outcome types -> Other (COMMENT) | Health care use |
| List Outcomes and Measurement tool | DD: DDS17 Benefit finding: Benefit Finding Scale (modified) Depression: PHQ-9 Anxiety: GAD7 Self-care: SDSCA Self-rated health: “In general, how would you rate your health at present?” Healthcare utilization: “In the past month, how many times have you visited a doctor or other health care professional?” Positive and negative affect: I-PANAS-SF How meaningful, personal, and distressing their writing exercise was: Essay Evaluation Measure Participants experiences and perceptions: Feedback Questionnaire |
| Location (city, country) | Australia |
| Healthcare setting and context/site of care | Online, Community-based |

| Author, Year | DuBois, 2016 |
| --- | --- |
| Aim of study | Primary aims were feasibility (measured by rates of positive psychology exercise completion) and acceptability (measured by participant ratings of exercise ease and utility). Explored the impact of the intervention by examining pre-post changes in positive psychological constructs, depression, anxiety, DD, diabetes self-care, and self-reported health behavior adherence. |
| Sample size | 15 |
| Population: Inclusion/exclusion criteria | Inclusion: English-speaking adult patients with T2D (meeting American Diabetes Association criteria, eg, glycated hemoglobin A (HbA) > 6.5% or fasting glucose > 126 mg/dL); suboptimal adherence (score < 15/18 on the MOS SAS items for medication, diet, and exercise).  Exclusion: cognitive impairment precluding consent or meaningful participation in the positive psychology exercises, assessed using a 6-item screen developed for research; lack of telephone access (given that the intervention was delivered via phone). |
| Population: Diabetes type | T2D |
| Population: Diabetes diagnosis duration (years, months) | N/R |
| Population: mental health disorder (if applicable) | N/A |
| Population: Mean age (year +/- SD) | 61.4 (7.0) |
| Population: Sex (N male and %) | 5 (41.7) |
| Study design | Pilot feasibility intervention (proof-of-concept).  One group, pre/post |
| Methods of study | Participants received a positive psychology manual, completed exercises (e.g., writing a gratitude letter, performing acts of kindness), and reviewed these activities by phone with a study trainer over a 12-week study period. In person or by telephone, the study interventionist reviewed the introductory portion of the manual and discussed the first exercise (gratitude for positive events) with the participant to increase understanding, alliance, and engagement with the intervention. In subsequent weeks, exercises were completed independently by participants and recorded in their treatment manual. In the final week, after exercise review, the interventionist and participant discussed future implementation and ways to incorporate the principles into daily life. Together, they also created a specific, written plan to perform positive psychological activities over the next 4 weeks, with the goal of maintaining use of these skills. |
| Delivery person(s) | Study trainer - not specified if health professional. |
| Study length AND  Start and end date of study | Study length: 12 weeks December 2013 - December 2014 |
| Study outcome timepoints | Baseline; 6 weeks; 12 weeks (post intervention) |
| Outcome types -> Process | Study feasibility, adherence, etc |
| Outcome types -> Patient reported | QoL, distress, anxiety, depression, knowledge, etc |
| List Outcomes and Measurement tool | Feasibility: Exercise completion   Acceptability/Impact: Optimism: 0–10 Likert scale Positive affect: 0–10 Likert scale Ease/utility of activity: 0-10  Optimism: Life Orientation Test-Revised Gratitude: Gratitude questionnaire-6 Anxiety and Depression: HADS DD: DDS Health-related QoL: NIH Patient-Reported Outcomes Measurement Information System physical function scale Diabetes self-care behaviors: SDSCA  Each outcome was also assessed via open-ended questions at 12 weeks |
| Location (city, country) | USA |
| Healthcare setting and context/site of care | Hospital (in-patient and out-patient) and clinic visits |

| Author, Year | Franco, 2018 |
| --- | --- |
| Aim of study | Describe web-based interventions for depression in individuals with diabetes and to discuss these studies’ procedures and findings in light of evidence from a wider range of interventions for depression and diabetes. |
| Sample size | 5 studies |
| Population: Inclusion/exclusion criteria | Inclusion and Exclusion: Eligible studies published in English or Spanish in a peer-reviewed journal between 1990 (coinciding with introduction of the World Wide Web in 1991) and 2017. Participants: Studies had to target adult participants (≥18 years) with a primary diagnosis of diabetes and comorbid depression. Depression was defined according to diagnostic criteria (Diagnostic and Statistical Manual of Psychiatric Disorders) or depressive symptomatology (on a validated self-report or clinician measure). Web-Based Interventions: The examined web-based interventions required the following components: program content (ie, psychoeducation and skills training guided by psychological theory); multimedia; provision of web-based activities; and a guided or unguided self-help approach. Eligible interventions had to target depression symptomatology with the specific intent of producing emotional, behavioral, and cognitive change. Study Design: Intervention studies with a repeated measures design, including randomized controlled trials and quasi experimental studies, were eligible. |
| Population: Diabetes type | Mixed |
| Population: Diabetes diagnosis duration (years, months) | N/R |
| Population: mental health disorder (if applicable) | N/A |
| Population: Mean age (year +/- SD) | N/R |
| Population: Sex (N male and %) | N/R |
| Study design | Review |
| Methods of study | A comprehensive literature search was conducted in PsycINFO and MEDLINE electronic databases. Studies were included when they met the following selection criteria: the study was available in a peer-reviewed journal mainly publishing studies written in either English or Spanish; the studied sample comprised individuals with diabetes; the intervention targeted depression symptomatology; the intervention was accessible via the internet; and the intervention was accessible via the internet with little or no clinician support. |
| Delivery person(s) | N/A |
| Study length AND  Start and end date of study | N/R |
| Study outcome timepoints | N/A |
| Outcome types -> Patient reported | QoL, distress, anxiety, depression, knowledge, etc |
| Outcome types -> Health | Glucose measures, body comp, cardio, etc |
| List Outcomes and Measurement tool | Study characteristics: type of study, sample size, measures Participants’ compliance: dropout percentage  Intervention efficacy: between-group effect size in depression and diabetes-related measures Intervention characteristics: delivery mode, psychotherapeutic approach, and research design  Sample characteristics: Sample size and medical diagnosis  Treatment characteristics: delivery format, therapeutic approach, therapist and peers support, and adherence management |
| Location (city, country) | N/A |
| Healthcare setting and context/site of care | N/A |

| Author, Year | Magee, 2021 |
| --- | --- |
| Aim of study | Examined the feasibility of co-delivering a mental health intervention with an evidence-based T2DM boot camp care management program. The preliminary impact of participation on symptom scores for depression and anxiety and A1C was also examined. |
| Sample size | 18 |
| Population: Inclusion/exclusion criteria | Inclusion: >18 years; diagnosis of T2DM with A1C >9%; screening scores between 10 and 19 on the PHQ-9 and/or on the (GAD-7) survey, scores consistent with moderate to moderately severe depression and/or anxiety; and English speaking  Exclusion: N/R |
| Population: Diabetes type | T2D |
| Population: Diabetes diagnosis duration (years, months) | N/R |
| Population: mental health disorder (if applicable) | moderate to moderately severe depression and/or anxiety |
| Population: Mean age (year +/- SD) | 50.7 (13.4) |
| Population: Sex (N male and %) | 3 (17) |
| Study design | pilot feasibility, open, non-randomized trial (one group pre/post) |
| Methods of study | Theory: CBT and MI  Participants in the DM-MH intervention concurrently received the 3-month Boot Camp intervention. This program diverges from standard diabetes self-management education in its level of intensity, frequency of touches with the participant, use of real-time blood glucose monitoring, offering of remote visits, and provision of T2DM medication management by the Boot Camp team. The mental health intervention component comprised 6 structured sessions. The intervention was initiated during the second one-on-one Boot Camp site visit. The mental health interventionist met with each participant individually for the initial mental health visit, during which a collaborative working relationship with each participant was established, an intervention plan was designed based on depression and anxiety screening results, and the mental health component of the intervention was initiated. Four subsequent mental health visits were then conducted remotely via telephone at 1 to 2 weekly intervals. The final booster behavioral session was delivered on site in conjunction with the final Boot Camp program visit. |
| Delivery person(s) | Clinical social worker trained by licensed clinical psychologist. |
| Study length AND  Start and end date of study | 12 weeks, N/R |
| Study outcome timepoints | Baseline; 12 weeks post intervention |
| Outcome types -> Process | study feasibility, adherence, etc |
| Outcome types -> Patient reported | QoL, distress, anxiety, depression, knowledge, etc |
| Outcome types -> Health | glucose measures, body comp, cardio, etc |
| List Outcomes and Measurement tool | baseline demographics: N/R clinical history: N/R A1C: N/R Depression symptoms: PHQ9 Anxiety symptoms: GAD-7 9-question post behavioral intervention evaluation asking them to rate their satisfaction with the intervention and to offer feedback on the techniques used and potential ways to improve the program. *post intervention only |
| Location (city, country) | Washington, DC, USA |
| Healthcare setting and context/site of care | Diabetes Boot Camp program at a large urban teaching hospital and medical center in Washington, DC. |

| Author, Year | Mochari-Greenberger, 2016 |
| --- | --- |
| Aim of study | Evaluated the feasibility and potential impact of a tele-behavioral healthcare program uniquely designed to treat comorbid behavioral health issues associated with diabetes to change participant depression, anxiety, and stress measures. Secondary aims were to determine whether the degree of change in depression, anxiety, or stress scores varied by participant demographic or clinical characteristics and evaluate changes in glucose self-monitoring frequency and glucose levels from program initiation to graduation. |
| Sample size | 466 |
| Population: Inclusion/exclusion criteria | Inclusion: documented diagnosis of having diabetes mellitus in combination with a recent change in medical status, such as an acute event (e.g., hospitalization) or a new medication regimen.  Exclusion: suicide attempt in the past 3 years; psychiatric hospitalization in the past year; current suicidal ideation or nonsuicidal self-injury; bipolar disorder, symptomatic in the past year or not medication stable; severe substance abuse disorder, substance intoxication or withdrawal, or high score on substance use disorder assessment; psychotic symptoms; borderline intellectual functioning; major neurocognitive disorder; domestic violence or homicidal ideation; end-stage illness |
| Population: Diabetes type | Mixed (type N/R) |
| Population: Diabetes diagnosis duration (years, months) | N/R |
| Population: mental health disorder (if applicable) | N/A |
| Population: Mean age (year +/- SD) | 56.8 (5.0) |
| Population: Sex (N male and %) | 207 (44) |
| Study design | Retrospective pre-post feasibility study. |
| Methods of study | AbilTo’s diabetes program was specifically designed to help individuals with diabetes understand and better manage depressive symptoms in support of medical compliance and positive lifestyle changes. The program was developed using evidence-based approaches and behavior change tools, including cognitive behavioral therapy, acceptance and commitment therapy, mindfulness, and motivational interviewing, and clinical guidelines to reduce depression, anxiety, and stress and to improve self-management and outcomes among people living with diabetes.  Program delivery occurred through telephone or secure video based on participant preference. The program was administered by a care team, which was made up of a behavioral health provider (licensed clinical social worker or equivalent [LCSW]) and a behavioral coach. The AbilTo diabetes program comprised an initial consultation and 15 sessions completed within an 8-week period. The initial consultation included a baseline interview and was conducted by an LCSW on the care team. The initial consultation was followed by eight sessions with an LCSW and seven sessions with a behavioral coach. During the course of the program, the LCSW and the behavioral coach participated in case conferences under the guidance of an LCSW clinical supervisor to review participant progress. The clinical supervisor also reviewed session notes on a weekly basis to ensure high quality and adherence to the treatment protocol. The protocol comprised modules targeted to specific skills and tailored to the individual participants’ needs and goals, drawing from the principles of cognitive behavioral therapy and other evidence-based behavioral health intervention strategies. |
| Delivery person(s) | Social worker and behavioural coach |
| Study length AND  Start and end date of study | Study length: 8 weeks August 1, 2014, and January 31, 2015 |
| Study outcome timepoints | Baseline to program graduation/8 weeks |
| Outcome types -> Patient reported | QoL, distress, anxiety, depression, knowledge, etc |
| Outcome types -> Health | Health (glucose measures, body comp, cardio, etc) |
| List Outcomes and Measurement tool | Depression, anxiety, and stress: Depression Anxiety Stress Scale 21 (DASS-21) Change in adherence to morning glucose self-testing: self-testing and documenting at least once weekly Change in morning blood glucose level: weekly average of morning glucose readings recorded by the participant using a point-of-care monitor; mg/dL |
| Location (city, country) | USA |
| Healthcare setting and context/site of care | N/A - digital healthcare provider |

| Author, Year | Murray, 2017 |
| --- | --- |
| Aim of study | To determine the effectiveness of a web-based self-management programme (HeLP-Diabetes) for people with T2D in improving glycaemic control and reducing diabetes-related distress. |
| Sample size | 374 |
| Population: Inclusion/exclusion criteria | Inclusion: aged ≥18, with T2DM, registered with participating general practices.  Exclusion: unable to provide informed consent; unable to use a computer due to severe mental or physical impairment; insufficient spoken or written English to use the intervention (operationalised as unable to consult without an interpreter); terminally ill with less than 12 months life expectancy; or were currently participating in a trial of an alternative self-management programme. |
| Population: Diabetes type | T2D |
| Population: Diabetes diagnosis duration (years, months) | Time since diagnosis (years), INT n (%) CON n (%) 0–4years: 70 (38%); 64 (34%)  5–9years: 55 (30%); 60 (32%)  10–14years: 40 (22%); 40 (21%)  15+ years: 18 (10%); 23 (12%) |
| Population: mental health disorder (if applicable) | N/A |
| Population: Mean age (year +/- SD) | Age at randomisation (years): INT: 64.9 (9.5): CON 64.7 (9.1) |
| Population: Sex (N male and %) | INT: 127 (69%); CON: 131 (69%) |
| Study design | Multicentre, two-arm individually randomised controlled trial. |
| Methods of study | Theory: Corbin and Strauss model The intervention consisted of facilitated access to HeLP-Diabetes. Facilitation consisted of an introductory training session with the practice nurse. HeLP-Diabetes was a theoretically informed web-based programme whose overall goals were to improve health outcomes and reduce diabetes-related distress. Content was designed to be accessible to people with a wide range of literacy and health literacy skills, with all essential content provided in both video and text. There were information sections on diabetes, how diabetes is treated, possible complications of diabetes, possible impacts of diabetes on relationships at home and at work, dealing with unusual situations like parties, holidays, travelling or shift work and what lifestyle modifications will improve health. There were sections addressing skills and behaviour change, including behaviour change modules on eating healthily, losing weight, being more physically active, smoking cessation, moderating alcohol consumption, managing medicines, glycaemic control and blood pressure control. Users could set the programme to send themselves reminder text messages or emails, and could specify the content and frequency of such reminders. The third strand of components focused on emotional wellbeing with self-help tools based on cognitive behavioural therapy and mindfulness. There were multiple personal stories (used with license from health talk online), and a moderated forum. Engagement with the programme was promoted through regular newsletters, emails and short message service containing updates on latest diabetes-related research or practice, seasonally relevant advice (eg, fasting during Ramadan, benefits of ‘influenza’ vaccinations), and links to specific relevant parts of the programme. Two or three prompts were sent each month, although users could opt-out of receiving them.   Comparator Given access to a simple information website, based on the information available on the website of the main UK diabetes charity (Diabetes UK) or National Health Service patient information website (NHS Choices). They received the same initial facilitation meeting as participants in the intervention group, in which they were shown how to log on, set a user name and password and how to use the website. |
| Delivery person(s) | Nurse facilitated but self-directed. |
| Study length AND  Start and end date of study | Study length: 12 months Recruitment: Sept 2013 to Dec 2014 |
| Study outcome timepoints | Baseline; 3 months, and 12 months post intervention |
| Outcome types -> Process | study feasibility, adherence, etc |
| Outcome types -> Patient reported | QoL, distress, anxiety, depression, knowledge, etc |
| Outcome types -> Health | glucose measures, body comp, cardio, etc |
| List Outcomes and Measurement tool | HbA1c  DD: PAID  SBP and DBP  BMI: body mass index total cholesterol and HDL (not fasting)  Completion of the ‘nine essential processes’ for effective management of diabetes, mandated by NHS England (weight, blood pressure, smoking status, measurement of serum creatinine, cholesterol and HbA1c, urinary albumin and assessment of eyes and feet) Depression and anxiety: HADS Diabetes-related self-efficacy: Diabetes Management Self-Efficacy Scale (DMSES) Satisfaction with treatment: Diabetes Satisfaction with Treatment Questionnaire status and change version (DTSQs and DTSQc). |
| Location (city, country) | England |
| Healthcare setting and context/site of care | 21 general practices in England with a mix of urban, suburban and rural practices. |

| Author, Year | Naik, 2012 |
| --- | --- |
| Aim of study | Examined the acceptability, feasibility and preliminary outcomes of a telephone-delivered behavioral coaching intervention for rural-dwelling older adults with uncontrolled diabetes and comorbid, clinically significant depressive symptoms. |
| Sample size | 8 |
| Population: Inclusion/exclusion criteria | Inclusion: rural-dwelling individuals with uncontrolled type 2 diabetes mellitus, as indicated by average HbA1c ≥7.5 and no single HbA1c marker <7.0 over the past year; met baseline depression screening requirements (i.e., PHQ-9 score ≥ 10). |
| Population: Diabetes type | Uncontrolled T2D |
| Population: Diabetes diagnosis duration (years, months) | years living with diabetes: 17.4 (7.39) |
| Population: mental health disorder (if applicable) | Depression |
| Population: Mean age (year +/- SD) | 62.1 (2.85) |
| Population: Sex (N male and %) | 6 (86) |
| Study design | Small open trial format to examine acceptability, feasibility. One group pre/post |
| Methods of study | The HOPE intervention consisted of ten 30–45 minute sessions delivered by telephone over a 12-week period. The overall objective of HOPE was to assist participants to improve their ability to self-manage their physical and emotional health. HOPE coaches sought to assist participants to prioritize their self-care needs, and to identify and resolve barriers to effective self-care. Participants learned and practiced skills that would help them reach the goals and action plans they set for themselves to improve their self-care. HOPE uses a structured patient workbook to guide participants and coaches. The workbook used a module-based approach where participants and coaches selected treatment goals and self-management skills that met the individual needs of the patient. The workbook was structured to facilitate behavioral change through collaborative goal-setting and action planning across physical and emotional health domains. It was ideographic by nature, allowing coaches to use structured information presented in physical and emotional health modules to stimulate conversation around issues of personal salience to patients, to educate patients on these issues and to build skills necessary to practice goals and action plans. Goal-setting and action planning components (e.g., worksheets to stimulate collaborative construction of high-quality goals and action plans) were incorporated throughout physical and emotional heath modules. The coaching model for the HOPE Program was based on the 5 As Model for coping with chronic illness. HOPE was structured around core concepts but also included a modular component that allowed coaches and patients to focus on physical and mental health difficulties of personal salience to the participant. The goal of this modular-based approach was to advance patient involvement and empowerment through active participation in the treatment process. |
| Delivery person(s) | Clinical psychology graduate intern, a developmental psychology postdoctoral fellow, a doctoral student in public health, and a post-bachelors psychology student. |
| Study length AND  Start and end date of study | Study length: 12 weeks N/R |
| Study outcome timepoints | Baseline; 12 weeks post intervention; 6 months follow up |
| Outcome types -> Patient reported | QoL, distress, anxiety, depression, knowledge, etc |
| Outcome types -> Health | Health (glucose measures, body comp, cardio, etc |
| List Outcomes and Measurement tool | Glycated Hemoglobin: HbA1c Depression: PHQ-9 DD: PAID |
| Location (city, country) | Houston, Texas, USA |
| Healthcare setting and context/site of care | Rural-dwelling |

| Author, Year | Naik, 2019 |
| --- | --- |
| Aim of study | Evaluated the effectiveness of HOPE for clinically significant improvements in depression and glycemic control compared with EUC—usual diabetes and depression care enhanced by a systems approach to screening for high-risk status. |
| Sample size | 225 |
| Population: Inclusion/exclusion criteria | Inclusion: uncontrolled diabetes who lived at least 20 miles from the main Veterans Health Administration hospital in Houston, Texas, or who received primary care services within a MEDVAMC satellite community-based clinic across Southeast Texas; clinically significant depression symptoms (score 10 on the PHQ-9); uncontrolled diabetes at baseline (HbA1c >7.5%)  Exclusion: absence of depression symptoms; a telephone-based coaching intervention would be inappropriate (i.e. the patient had severe cognitive impairment or mental health condition, hearing or visual impairment, or active suicidal ideation); presence of significant hypoglycemic events or substance abuse |
| Population: Diabetes type | N/R - uncontrolled diabetes of unknown type |
| Population: Diabetes diagnosis duration (years, months) | N/R |
| Population: mental health disorder (if applicable) | Depression (score 10 on the PHQ-9) |
| Population: Mean age (year +/- SD) | N/R as mean (SD) Age ≥65 y, N (%): 104 (46.2) |
| Population: Sex (N male and %) | 202 (89.8) |
| Study design | Randomised controlled trial |
| Methods of study | HOPE Participants: Received 9 coaching sessions with a trained health professional: biweekly (for 30-40 minutes) from months 1 to 3 and monthly (for 15 minutes) from months 4 to 6. Twenty-four trained health professionals or coaches (18 female) included psychologists (n = 16), nurses (n = 5), pharmacists (n = 2), and social workers (n = 1). Most (n = 18) were at the MEDVAMC; 6 were at a Veterans Health Administration community-based clinic. Patients and coaches used workbooks that guided telephone conversations and allowed patients to define and track their progress. Primary care physicians received notifications of their patients’ participation, HbA1c results, and PHQ-9 questionnaire outcomes via secure electronic messaging; however, they received no formal training related to the HOPE intervention components. During the first 2 patient sessions, HOPE coaches focused on building rapport, introducing and clarifying values, collaboratively setting initial goals, identifying potential skill sets to address goals, and empowering patients to advocate for their health through active communication with their clinicians. For sessions 3 through 6, participants focused on discrete skill modules (increasing pleasant activities, using thoughts to improve wellness, diet, physical activity, medication management, and relaxation) customized to meet their diabetes and depression goals. Sessions 7 through 9 focused on maintenance skills (reviewing action plans and overcoming barriers). Skills emphasized in the modules were designed to improve diabetes- and depression-related outcomes simultaneously. The HOPE modules stressed the importance of the coach-patient relationship as critical to improvement in participant physical and/or emotional self-management. During months 7 to 12, participants received usual primary care without contact from HOPE coaches.  EUC: In addition to usual care, EUC participants were informed about their high-risk status (uncontrolled diabetes status and clinically significant depression symptoms) and were given related educational materials. Study assessments were conducted for EUC participants via telephone, and educational materials were mailed. Participants were encouraged to address these results with their primary care clinician. |
| Delivery person(s) | psychologists, nurses, pharmacists, social workers |
| Study length AND  Start and end date of study | Study length: 12 months, with first 6 months active intervention Study conducted: November 1, 2012, through June 24, 2016. Data collection was completed on December 6, 2016, and final analyses were completed by January 25, 2018 |
| Study outcome timepoints | Baseline; 6 months post intervention; and 12 months |
| Outcome types -> Patient reported | QoL, distress, anxiety, depression, knowledge, etc |
| Outcome types -> Health | glucose measures, body comp, cardio, etc |
| Outcome types -> Other | cost, provider experience, etc |
| Outcome types -> Other (COMMENT) | health care use |
| List Outcomes and Measurement tool | Change in glycemic control: HbA1c  Depression symptoms: PHQ-9 DD: PAID Questionnaire Worry/anxiety: Penn State Worry Questionnaire Goal-Setting Evaluation Tool for Diabetes Health care use Self-efficacy Comorbid physical and mental conditions |
| Location (city, country) | Texas, USA |
| Healthcare setting and context/site of care | MEDVAMC and 6 affiliated community-based outpatient clinics across Southeast Texas |

| Author, Year | Newby, 2017 |
| --- | --- |
| Aim of study | Examined the efficacy of a generic 6-lesson iCBT delivered over 10 weeks in people with MDD and DM. |
| Sample size | 91 |
| Population: Inclusion/exclusion criteria | Inclusion: Australian resident; age ≥18 years; fluent in English; access to a computer and Internet; self-reported diagnosis of T1 or T2DM, meet criteria for MDD according to telephone-administered diagnostic interview, and provide personal and GP contact details.   Exclusion: self-reported diagnosis of bipolar affective disorder, psychotic disorder or substance use disorder, or were taking antipsychotics or benzodiazepines; commenced CBT in the past month, or changed antidepressant medication in the past 2 months; score of either <5 (normal range) or >23 (very severe) on the patient health questionnaire-9 (PHQ-9); those identified as being at significant risk of suicide or deliberate self-harm in the telephone risk assessment. |
| Population: Diabetes type | Mixed (T1D, T2D) |
| Population: Diabetes diagnosis duration (years, months) | N/R |
| Population: mental health disorder (if applicable) | Major depressive disorder |
| Population: Mean age (year +/- SD) | 46.7 (12.6) |
| Population: Sex (N male and %) | 26 (29) |
| Study design | Randomised controlled trial. |
| Methods of study | Theory: CBT Completed 6 automated cartoon-style Web-based lessons teaching CBT skills (ex. behavioral activation) over 10 weeks, with a minimum wait-time of 5 days between lessons. Participants downloaded a “homework” document which included practical assignments (ex. thought monitoring) after each lesson, and had access to extra resources, frequently asked questions, and recovery stories of former participants. Automated reminder emails were also sent to participants when lessons became available. Participants in the iCBT group were able to continue to receive usual care from their health services during the intervention period.  The treatment as usual group received the iCBT intervention after 10 weeks. |
| Delivery person(s) | None – automated |
| Study length AND  Start and end date of study | Study length: 10 weeks  Sept 2013 - June 2015 |
| Study outcome timepoints | Baseline; 11 weeks post intervention; and 3-month follow-up (for intervention group only) |
| Outcome types -> Process | Study feasibility, adherence, etc |
| Outcome types -> Patient reported | QoL, distress, anxiety, depression, knowledge, etc |
| List Outcomes and Measurement tool | Depression: PHQ-9 Glycemic control: HbA1C DD: PAID Psychological distress: K10 Mental & physical well-being: SF-12 Anxiety: GAD-7 Somatic symptom severity: PHQ-15 relationships questionnaire (to assess attachment style): the fantastic checklist to assess specific lifestyle behaviours such as smoking status and alcohol use  Intervention acceptability and satisfaction: "credibility or expectancy questionnaire" |
| Location (city, country) | New South Wales, Australia |
| Healthcare setting and context/site of care | Online/virtual (There were no face-to-face components of the study) Community |

| Author, Year | Nobis, 2015 |
| --- | --- |
| Aim of study | Evaluated the efficacy of a guided web-based intervention in reducing depression in adults with T1D and T2D. |
| Sample size | 260 |
| Population: Inclusion/exclusion criteria | Inclusion: German-speaking adults with T1D and T2D and those with both moderate and high depressive symptoms (Center for Epidemiologic Studies Depression Scale [CES-D] ≥23).  Exclusion: suicide risk; current psychotherapeutic treatment or presence on a waiting list for such treatment. |
| Population: Diabetes type | Mixed (T1D and T2D) |
| Population: Diabetes diagnosis duration (years, months) | Diabetes duration for TOTAL, n (%) Duration of diabetes, 3–12 months: 18 (7) Duration of diabetes, 1–10 years: 119 (46) Duration of diabetes, >10 years: 119 (46) |
| Population: mental health disorder (if applicable) | Depressive symptoms |
| Population: Mean age (year +/- SD) | 51 (12) |
| Population: Sex (N male and %) | 94 (37) |
| Study design | Randomised controlled trial. |
| Methods of study | The GET.ON M.E.D. intervention consisted of six consecutive sessions, with the opportunity of two additional sessions addressing weight management and improving healthy sleeping. They were advised to complete one session per week; each session lasted 45 min. One month after the end of the intervention, participants were offered an optional booster session. The intervention GET.ON M.E.D. was based on two core evidence-based elements: systematic behavioral activation and problem solving. Diabetes-specific themes were an essential part of each session, covering the link between diabetes and depression, worrying about diabetes problems, diabetes and sexuality, physical activity, and communication with general practitioners. Coaches (graduate students or psychologists) gave personalized feedback by e-mail within 48 h of each session. Each coach was supervised by an experienced clinical psychologist. The participants had the opportunity to receive daily standardized text messages, with the aim of supporting participants to reach intervention goals and to implement behavioral change strategies. If a participant showed no activity within 7 days, the coach sent an e-mail and offered assistance. If there was no contact within 1 week, the participant received a phone call. Participants were asked about current obstacles and encouraged to proceed with the intervention.  Control Group Received access to an online psychoeducational program, based on the German S3-Guideline/National Disease Management Guideline for Unipolar Depression. |
| Delivery person(s) | "Coaches" - graduate students or psychologists |
| Study length AND  Start and end date of study | Study length: 8 weeks Recruitment March 2013 to January 2014 |
| Study outcome timepoints | Baseline (T1); post treatment (8 weeks, T2); 6-months follow-up (6-MFU) |
| Outcome types -> Process | Study feasibility, adherence, etc |
| Outcome types -> Patient reported | QoL, distress, anxiety, depression, knowledge, etc |
| Outcome types -> Health | Glucose measures, body comp, cardio, etc |
| Outcome types -> Other | Cost, provider experience, etc |
| List Outcomes and Measurement tool | Depressive symptom severity: German version of the CES-D Depressive symptoms: HADS DD: PAID  Coping with diabetes: German version of the AADQ Diabetes self-management: DSMQ User Satisfaction. adapted, eight-item version of the German CSQ-8 Glycaemic control: HbA1c values Physical and mental functioning: SF-12 Cost-utility analysis: QALYs |
| Location (city, country) | Germany |
| Healthcare setting and context/site of care | N/A - online, not recruited from specific healthcare setting |

| Author, Year | Orman, 2016 |
| --- | --- |
| Aim of study | Examined whether a diabetes-specific online module, integrated into an existing e-mental health program, is acceptable to patients and helps to improve psychological wellbeing. |
| Sample size | 35 |
| Population: Inclusion/exclusion criteria | Inclusion: diagnosed with T1D or T2D by a GP or endocrinologist; experienced at least mild depressive symptoms (as measured by the PHQ-9); aged 18–75 years; an Australian resident; access to the internet via both mobile phone and computer; valid email address.  Exclusion: self-reported psychotic symptoms (as assessed by the psychosis screening questionnaire) or severe suicidal ideation (item 9 on the PHQ-9) |
| Population: Diabetes type | Mixed (T1D and T2D) |
| Population: Diabetes diagnosis duration (years, months) | N/R |
| Population: mental health disorder (if applicable) | mild depressive symptoms |
| Population: Mean age (year +/- SD) | 47.6 years (range 26–74 years) |
| Population: Sex (N male and %) | 14 (40) |
| Study design | Pre/post mixed methods pilot study. |
| Methods of study | First stage Qualitative data were collected from focus groups and interviews conducted with GPs and consumers to determine the perceived need for (GPs and consumers) and desirable content of (consumers) a diabetes-specific online module.   Second stage Theory: ACT and CBT Clinical content for the online module was developed by a team of mental health researchers at the Black Dog Institute in Sydney, including the authors, using information from the first stage. The module blends skills from traditional CBT and ACT, both of which are evidence-based treatments for mental health symptoms generally, and in diabetes patients, specifically. Consistent with existing myCompass modules, the new module titled “Doing what really counts” consists of three 10-minute sessions, each of which includes written information, interactive skill-building exercises, vignettes and home tasks (for in-between sessions). The module steps users through identification of important life domains and values, goal-setting techniques and identification and management of obstacles to goal achievement. Throughout the module, users are encouraged to consider diabetes and physical health as one of several important life domains and to pursue valued activities that create rich and fulfilling lives.   Third stage The new module was pilot-tested in a group of 35 patients with diabetes. Participants completed the standardised outcome measures at baseline (before using the online module) and at four weeks. |
| Delivery person(s) | self-led N/A. |
| Study length AND  Start and end date of study | Study length: 4 weeks N/R |
| Study outcome timepoints | Baseline; 4 weeks |
| Outcome types -> Process | Study feasibility, adherence, etc |
| Outcome types -> Patient reported | QoL, distress, anxiety, depression, knowledge, etc |
| Outcome types -> Health | Glucose measures, body comp, cardio, etc |
| List Outcomes and Measurement tool | Qualitative data focus groups and interviews conducted with GPs and consumers to determine the perceived need for (GPs and consumers) and desirable content of (consumers) a diabetes-specific online module. Participants also rated their experience with the module using a set of eight study-specific items  Quantitative data • depressive and anxiety symptoms: PHQ-9 and GAD-7 • DD: PAID • perceived confidence in managing diabetes and mental health: SEDS and MHSES • daily self-management of diabetes: DSCA • self-reported glycaemic control: HHS • work and social functioning: WSAS |
| Location (city, country) | New South Wales, Australia |
| Healthcare setting and context/site of care | online N/A |

| Author, Year | Piette, 2011 |
| --- | --- |
| Aim of study | Evaluated the impact of telephone-delivered CBT targeting patient management of depressive symptoms, physical activity, and diabetes-related outcomes. |
| Sample size | 291 |
| Population: Inclusion/exclusion criteria | Inclusion: ≥21 years; T2DM; prescription of antihyperglycemic medication  Exclusion: PHQ-9 depression score <11; not using antihyperglycemic medication; diagnosed with bipolar disease/schizophrenia; active treatment for other serious medical condition (e.g., heart failure, COPD, ESRD); if using anti-depressants and reported a change in medication/physician prescribing in 30 days prior to enrollment; unable to walk one block/10 minutes without rest |
| Population: Diabetes type | T2D |
| Population: Diabetes diagnosis duration (years, months) | N/R |
| Population: mental health disorder (if applicable) | Depression (Moderate+ PHQ-9 score >11) |
| Population: Mean age (year +/- SD) | N/R |
| Population: Sex (N male and %) | N/R |
| Study design | Randomised controlled trial. |
| Methods of study | Theory: CBT 12-month telephone CBT programme: included an initial intensive phase of 12-weekly sessions followed by 9-monthly booster sessions. At first, CBT focused exclusively on patients’ depressive symptoms; after five sessions, nurse counsellors introduced concepts related to a pedometer-based walking programme, and the links between depression, physical activity, and diabetes outcomes. Sessions were guided by a week-by-week manual. While intervention nurses were trained to work relatively independently, several aspects of the protocol ensured communication with patients’ primary care teams. Nurses were supported by CBT training, supervision.  Usual care included a copy of the Feeling Good Handbook—a self-help book based on CBT for depression, National Institute of Mental Health educational materials about depression, educational materials about walking and diabetes, and a list of local resources for depression. |
| Delivery person(s) | RN (psychiatric/primary care training). |
| Study length AND  Start and end date of study | Study length: 12 months Recruitment March 2006-November 2008 |
| Study outcome timepoints | Baseline; 12 months post intervention |
| Outcome types -> Patient reported | QoL, distress, anxiety, depression, knowledge, etc |
| Outcome types -> Health | Glucose measures, body comp, cardio, etc |
| List Outcomes and Measurement tool | A1C: the DCA2000 point-of-care analyzer SBP and DBP: Omron automatic monitor with a repeat measurement in the arm with the highest pressure after several minutes of rest Step count: Omron HJ-720 ITC pedometer Depression: BDI Patients’ coping: Brief Cope Perceived self-efficacy for physical activity, diet: Perceived Competence Scale Adherence to antihyperglycaemic medication: Morisky medication adherence scale Patients’ beliefs about the benefits and potential negative consequences of diabetes medications: Beliefs about Medications Questionnaire Patients’ health-related quality of life: SF-12 Patients’ clinical complexity at baseline: Vector Control Model of Complexity |
| Location (city, country) | Michigan, USA |
| Healthcare setting and context/site of care | Community-based non-profit; university healthcare system; VA healthcare system (teaching sites of affiliated medical schools). |

| Author, Year | van Bastelaar, 2011 |
| --- | --- |
| Aim of study | Tested the effectiveness of DG.nl in a randomized controlled trial. |
| Sample size | 255 |
| Population: Inclusion/exclusion criteria | Inclusion: adult diabetic patients with a score of ≥ 16 on the CES-D; have an e-mail address; access to the Internet.  Exclusion: history of suicide attempt(s) or current suicidal ideation; bipolar depression or psychotic disorder; pregnancy; and recent loss of a significant other (>6 months ago). |
| Population: Diabetes type | Mixed (T1D & T2D) 55% T2D. |
| Population: Diabetes diagnosis duration (years, months) | Duration of diabetes, years 14(12). |
| Population: mental health disorder (if applicable) | Depression: score of ≥16 on CES-D. |
| Population: Mean age (year +/- SD) | 50 (12) |
| Population: Sex (N male and %) | 100 (39) |
| Study design | Randomised controlled trial. |
| Methods of study | Theory: CBT Participants individually went through eight consecutive lessons that provided written and spoken information and videos of depressed diabetic patients explaining how they learned from the course. Coaches (certified health psychologists) provided feedback on homework assignments less than or equal to 3 working days. Feedback was to a large degree standardized and consisted of a concise, constructive reply on the CBT techniques, meant to help patients understand and apply the CBT skills in daily practice. In case homework was not received, patients were sent reminders after 1 week and after 2 weeks. If no reply was received less than or equal to 3 weeks, participants received an e-mail stating we had to assume that they were no longer interested in the intervention, and were invited to fill out the post-measurement. However, if still interested, they were invited to re-enter the course.  Waiting list. Completed measurements 8 weeks post assessment and 12 weeks after randomization (1-month follow-up assessment). After this 12-week waiting period, patients received a password that allowed them to log in to the Web-based intervention, if they still had elevated depressive symptoms (CES-D ≥ 16). |
| Delivery person(s) | Coaches (certified health psychologists). |
| Study length AND  Start and end date of study | Study length: N/R Recruited from July 2008 through September 2009. |
| Study outcome timepoints | Baseline; post intervention; and 1 month follow-up. |
| Outcome types -> Patient reported | QoL, distress, anxiety, depression, knowledge, etc |
| Outcome types -> Health | Glucose measures, body comp, cardio, etc |
| List Outcomes and Measurement tool | Depression: CES-D Diabetes-specific emotional distress: Dutch version of PAID glycemic control: A1C glycosylated hemoglobin |
| Location (city, country) | Amsterdam, the Netherlands |
| Healthcare setting and context/site of care | Online |

| Author, Year | van der Feltz-Cornelis, 2013 |
| --- | --- |
| Aim of study | Addressed what treatments of comorbid depression in diabetes mellitus can positively impact diabetes disease control, and what evidence for this view has emerged since 2010, with a focus on psychotherapeutic and pharmacotherapeutic versus E-health or M-health interventions? |
| Sample size | N/R |
| Population: Inclusion/exclusion criteria | N/R |
| Population: Diabetes type | N/R |
| Population: Diabetes diagnosis duration (years, months) | N/R |
| Population: mental health disorder (if applicable) | N/A |
| Population: Mean age (year +/- SD) | N/R |
| Population: Sex (N male and %) | N/R |
| Study design | Literature Review |
| Methods of study | N/R |
| Delivery person(s) | N/A |
| Study length AND  Start and end date of study | N/R |
| Study outcome timepoints | N/R |
| Outcome types -> Patient reported | QoL, distress, anxiety, depression, knowledge, etc |
| Outcome types -> Health | Glucose measures, body comp, cardio, etc |
| List Outcomes and Measurement tool | N/R |
| Location (city, country) | N/R |
| Healthcare setting and context/site of care | N/R |

| Author, Year | Wu, 2018 |
| --- | --- |
| Aim of study | Compared 6-month outcomes of a technology-facilitated care model with a usual care model and a supported care model that involved team-based collaborative depression care for safety-net primary care adult patients with type 2 diabetes. |
| Sample size | 1406 |
| Population: Inclusion/exclusion criteria | Inclusion: ≥18 years; diagnosed with T2D; working phone number; spoke English or Spanish; could read and understand the consent form  Exclusion: Patients with baseline possible suicidal ideation; cognitive impairment; alcohol abuse; recent lithium or antipsychotic medication |
| Population: Diabetes type | T2D |
| Population: Diabetes diagnosis duration (years, months) | Age at onset of diabetes, mean (SD): UC 45.20 (10.52); SC: 41.84 (10.19); TC: 42.32 (9.84) |
| Population: mental health disorder (if applicable) | N/A |
| Population: Mean age (year +/- SD) | UC: 55.15 (9.21); SC: 51.92 (9.29); TC: 52.63 (8.74) |
| Population: Sex (N male and %) | UC: 123 (30); SC: 190 (41); TC: 166 (39) |
| Study design | Clinical controlled trial/comparative effectiveness study with quasi-experimental design. |
| Methods of study | During the first 6 months, the UC group received usual primary care, whereas the SC group received DMP-supported depression care, and the TC group received the ATA application in the DMP setting. After 6 months, all SC and TC patients were transferred back to their usual primary care, although the ATA calls were continued for the full 12 months.  Supported Care Model The SC model used the diabetes DMP team (comprising nurse care managers, nurse practitioners, and a consulting or supervising physician) to deliver depression care. SC diabetes care management was designed to proactively identify, risk stratify, and treat patients using clinical protocols that emphasized patient empowerment. The DMP was nurse driven and physician supervised and used structured approaches and protocols; in these programs, nurses delivered the majority of the diabetes care. The approaches included a patient-signed commitment to take an active role in his or her diabetes care, case management, PCP designation, group patient education, self-management support, and care coordination. The diabetes-specific management was provided initially via in-person visits, with follow-up primarily via telephone visits. The DMP included a homegrown, Web-based, interactive chronic DMR system to support clinical assessment and decisions. The DMP was designed for limited-time care management (typically 6 months), after which patients were transferred back to their primary medical providers. The SC program also designated a social worker to provide problem-solving therapy, an evidence-based depression treatment.  Technology-Facilitated Care Model The TC model also operated in a DMP clinic setting with a DMR and supplemental depression care based on the LACDHS depression care protocol and treatment guideline. The TC model, however, was designed to assist time-pressured clinical social workers and medical and nursing providers by using an ATA system to routinely screen and monitor patient depression symptoms and treatment adherence and communicate the results to providers. The ATA system was linked with the DMR to automatically trigger depression care management calls on a predetermined calendar schedule. The call contents were individually tailored, driven by a preprogrammed algorithm that scanned patient medical records and call histories to determine applicable questions. There were two main ATA call scripts: one for screening and one for monitoring. The screening calls were for people who had no prior history of depression or who had been clear of a depression diagnosis for at least 6 months. The monitoring calls were for depressed patients; the monitoring calls addressed all four categories and administered PHQ-9. The calls were low intensity (ie, one call every month for monitoring or every 3 months for screening based on each patient’s depression condition) to balance information need and patient burden. The patient-reported ATA data were tethered to the DMR, which in the TC model was enhanced by clinical decision support software for provider collaborative communication. The decision support software automatically generated task reminders and alerts based on the patient records in the DMR and the assessment data; the reminders and alerts prompted DMP providers to follow up with specific patients in need of care. Task reminders included structured, radio-button lists of potential care management actions with the option of free text to support evidence-based practices and to ease providers’ documentation burden. |
| Delivery person(s) | Nurse care managers, nurse practitioners, and a consulting or supervising physician, social worker. |
| Study length AND  Start and end date of study | Study length: 12 months (the study occurred from 2011 to 2013) Enrollment period April 2011 to May 2012 |
| Study outcome timepoints | Baseline; 6 months; 12 months; and 18 months |
| Outcome types -> Process | Study feasibility, adherence, etc |
| Outcome types -> Patient reported | QoL, distress, anxiety, depression, knowledge, etc |
| Outcome types -> Health | Glucose measures, body comp, cardio, etc |
| Outcome types -> Other | Cost, provider experience, etc |
| Outcome types -> Other (COMMENT) | Cost |
| List Outcomes and Measurement tool | Depression; PHQ-9 and depression remission Diabetes: HbA1c, total cholesterol, diabetes self-care, exercise PROMs: SF-12 physical score, SF-12 mental score, Sheehan Disability Scale, Satisfaction with diabetes care  Satisfaction with care for emotional problems Patient engagement: each patient’s ATA call response rates (percentage of completed or incomplete calls divided by percentage of automated call attempts). Barriers to patient engagement: patients with incomplete calls were followed up with a human call to determine reasons for failure to contact.  ATA call acceptance  ATA call completion rates Medical care costs and utilization: LACDHS electronic medical services records  Intervention costs: actual budget-based costs (not charges) |
| Location (city, country) | LA, California, USA |
| Healthcare setting and context/site of care | Hospital-based outpatient clinic and a community clinic. |

| Author, Year | Yap, 2021 |
| --- | --- |
| Aim of study | Synthesised the best available evidence concerning the effectiveness of TBPIs on DD, self-efficacy, HRQoL and HbA1c level among adults with T2DM. The review question was ‘what is the effectiveness of TBPIs on DD, self-efficacy, HRQoL, and HbA1c level of individuals with T2DM?’ |
| Sample size | 20 studies |
| Population: Inclusion/exclusion criteria | Inclusion: at least 18 years old and with the diagnosis of T2DM; tested TBPIs (such as MI, behavioural therapy and CBT); compared TBPIs with usual care, enhanced usual care, waiting list and/or attentional control groups; measured at least one of these outcomes: DD, self-efficacy, HRQoL or HbA1c levels with validated measuring tools; used randomised controlled trials that were reported in English from 2010 to 2020; interventions delivered by healthcare providers and comprised more than 50% in-person sessions  Exclusion: studies with self-help groups, peer-delivered interventions or general education |
| Population: Diabetes type | T2D |
| Population: Diabetes diagnosis duration (years, months) | N/A |
| Population: mental health disorder (if applicable) | N/A |
| Population: Mean age (year +/- SD) | Overall N/R |
| Population: Sex (N male and %) | Overall N/R |
| Study design | Systematic Review and Meta-Analysis |
| Methods of study | A search of eleven databases was conducted to identify randomised controlled trials that examined the effects of technology-based psychosocial interventions on the outcomes. Randomised controlled trials reported in English from 2010 to 2020 were included. Selection of studies, quality appraisal, and data extraction were conducted by two reviewers independently. Meta-analyses, subgroup analyses and sensitivity analysis were performed using Review Manager. Intervention effects was measured using standardise mean difference. |
| Delivery person(s) | N/A |
| Study length AND  Start and end date of study | N/R |
| Study outcome timepoints | N/A |
| Outcome types -> Patient reported | QoL, distress, anxiety, depression, knowledge, etc |
| Outcome types -> Health | glucose measures, body comp, cardio, etc |
| List Outcomes and Measurement tool | DD, self-efficacy, HRQoL or HbA1c levels |
| Location (city, country) | N/A |
| Healthcare setting and context/site of care | N/A |

**Legend**

AADQ: Acceptance and Action Diabetes Questionnaire

ACT: Acceptance and Commitment-based Therapy

ATA: automated telephone assessment

BDI: Beck Depression Inventory

BFW: Benefit-finding writing

BMI: body mass index

CBT: cognitive behavioural therapy

CCT: Controlled clinical trial

CON: Control

CSQ: Client Satisfaction Questionnaire

CW: active control condition of online writing about the use of time

DASS-21: Depression, anxiety, and stress: Depression Anxiety Stress Scale 21

DBP: diastolic blood pressure

DD: diabetes distress

DDS: Diabetes Distress Scale

DES: Diabetes Empowerment Scale

DG.nl: Diabetergestemd.nl

DM-MH: diabetes and mental health co-management

DMP: diabetes disease management program

DMR: disease management registry

DMSES: Diabetes Management Self-Efficacy Scale

DSCA: Diabetes Self-Care Activities measure

DSMQ: Diabetes Self-Management Questionnaire

DTSQs and DTSQc: Diabetes Satisfaction with Treatment Questionnaire status and change version

EAT–26: Eating Attitudes Test

ESRD: end-stage renal disease

EUC: enhanced usual care

FoP-Q-SF: Fear of progression

GAD-7: Generalized Anxiety Disorder – 7

GP: general practitioner

HADS: Hospital Anxiety and Depression Scale

HbA1c: glycated haemoglobin

HeLP-Diabetes: Healthy Living for People with Diabetes

HHS: Hyperglycaemia and Hypoglycaemia Scale

HOPE: Healthy Outcomes Through Patient Empowerment

HRQoL: health-related quality of life

INT: Intervention

I-PANAS-SF: International Positive and Negative Affect Schedule Short Form

K10: Kessler psychological distress scale

LACDHS: Los Angeles County Department of Health Services

LCSW: Licensed clinical social worker or equivalent

MDD: major depressive disorder

MEDVAMC: Michael E. DeBakey VA Medical Center

MHSES: Mental Health Self-Efficacy Scale

MI: motivational interviewing

MOS SAS: Medical Outcomes Study Specific Adherence Scale

MSQ: Mini Sleep Questionnaire

N/R: not reported

PAID: Problem Areas In Diabetes

PHQ-9: Patient Health Questionnaire − 9

PSQ: Psychosis Screening Questionnaire

QALYs: Quality-adjusted life-years

SBP: systolic blood pressure

SC: supported care

SCIR: Self-Care Inventory-revised

SDSCA: Summary of Diabetes Self-Care Activities

SEDS: Self-Efficacy for Diabetes Scale

SF-12: Short Form Health Survey

SMA: Shared medical appointment

SMP-T2D: Self-Management Profile for Type 2 Diabetes

T1D: Type 1 diabetes

T2DM: Type 2 diabetes mellitus

TBPIs: technology-based psychosocial interventions

TC: technology-facilitated care

UC: usual care

WSAS: Work and Social Adjustment Scale

YAs: young adults
